# SMC5/6 complex-mediated SUMOylation stimulates DNA–protein cross-link repair in Arabidopsis

**DOI:** 10.1093/plcell/koad020

**Published:** 2023-01-27

**Authors:** Eva Dvořák Tomaštíková, Klara Prochazkova, Fen Yang, Jitka Jemelkova, Andreas Finke, Annika Dorn, Mahmoud Said, Holger Puchta, Ales Pecinka

**Affiliations:** Institute of Experimental Botany of the Czech Academy of Sciences, Centre of Plant Structural and Functional Genomics, Šlechtitelů 31, 77900 Olomouc, Czech Republic; Institute of Experimental Botany of the Czech Academy of Sciences, Centre of Plant Structural and Functional Genomics, Šlechtitelů 31, 77900 Olomouc, Czech Republic; Institute of Experimental Botany of the Czech Academy of Sciences, Centre of Plant Structural and Functional Genomics, Šlechtitelů 31, 77900 Olomouc, Czech Republic; Department of Cell Biology and Genetics, Faculty of Science, Palacký University, Šlechtitelů 27, 77900 Olomouc, Czech Republic; Institute of Experimental Botany of the Czech Academy of Sciences, Centre of Plant Structural and Functional Genomics, Šlechtitelů 31, 77900 Olomouc, Czech Republic; Functional Genomics and Proteomics, National Centre for Biomolecular Research (NCBR), Faculty of Science, Masaryk University, Kamenice 5, 62500 Brno, Czech Republic; Department of Plant Breeding and Genetics, Max Planck Institute for Plant Breeding Research, Carl-von-Linné-Weg 10, 50829 Cologne, Germany; Botanical Institute, Molecular Biology and Biochemistry, Karlsruhe Institute of Technology, Fritz-Haber-Weg 4, Karlsruhe, 76131, Germany; Institute of Experimental Botany of the Czech Academy of Sciences, Centre of Plant Structural and Functional Genomics, Šlechtitelů 31, 77900 Olomouc, Czech Republic; Field Crops Research Institute, Agricultural Research Centre, 9 Gamma Street, Giza, 12619, Cairo, Egypt; Botanical Institute, Molecular Biology and Biochemistry, Karlsruhe Institute of Technology, Fritz-Haber-Weg 4, Karlsruhe, 76131, Germany; Institute of Experimental Botany of the Czech Academy of Sciences, Centre of Plant Structural and Functional Genomics, Šlechtitelů 31, 77900 Olomouc, Czech Republic; Department of Cell Biology and Genetics, Faculty of Science, Palacký University, Šlechtitelů 27, 77900 Olomouc, Czech Republic

## Abstract

DNA–protein cross-links (DPCs) are highly toxic DNA lesions consisting of proteins covalently attached to chromosomal DNA. Unrepaired DPCs physically block DNA replication and transcription. Three DPC repair pathways have been identified in Arabidopsis (*Arabidopsis thaliana*) to date: the endonucleolytic cleavage of DNA by the structure-specific endonuclease MUS81; proteolytic degradation of the crosslinked protein by the metalloprotease WSS1A; and cleavage of the cross-link phosphodiester bonds by the tyrosyl phosphodiesterases TDP1 and TDP2. Here we describe the evolutionary conserved STRUCTURAL MAINTENANCE OF CHROMOSOMEs SMC5/6 complex as a crucial component involved in DPC repair. We identified multiple alleles of the SMC5/6 complex core subunit gene *SMC6B* via a forward-directed genetic screen designed to identify the factors involved in the repair of DPCs induced by the cytidine analog zebularine. We monitored plant growth and cell death in response to DPC-inducing chemicals, which revealed that the SMC5/6 complex is essential for the repair of several types of DPCs. Genetic interaction and sensitivity assays showed that the SMC5/6 complex works in parallel to the endonucleolytic and proteolytic pathways. The repair of zebularine-induced DPCs was associated with SMC5/6-dependent SUMOylation of the damage sites. Thus, we present the SMC5/6 complex as an important factor in plant DPC repair.

## Teaser

The SMC5/6 complex is a crucial component involved in the repair of highly toxic DNA–protein cross-links via a unique SUMO-dependent pathway.

**IN A NUTSHELL**

**Background:** Cellular DNA is constantly damaged by various internal and external factors that eventually lead to mutations, reduced growth or even death. To ensure genome stability, organisms have evolved sophisticated and intricate DNA repair systems. We understand how cells remove some types of DNA damage, but the mechanisms of detoxification from other types of damage remain poorly characterized. For example, DNA–protein cross-links, i.e. proteins covalently attached to DNA molecule, hinder the essential processes of replication and transcription.

**Question:** Our aim is to identify molecular factors protecting plants from toxic DNA–protein cross-links. We set up a forward-directed genetic screen to identify mutants hypersensitive to the cytidine analog zebularine, which cross-links DNA METHYLTRANSFERASE1 (MET1) protein to the 45S rDNA repeats, and characterized the first candidate.

**Findings:** We mapped *HYPERSENSITIVE TO ZEBULARINE 1* (*HZE1*) candidate as *SMC6B*, a core component of the structural maintenance of chromosomes 5/6 (SMC5/6) complex. HZE1 plays a key role in DNA–protein cross-link repair as it is needed for the repair of different classes of cross-links. We also showed that the SMC5/6 complex acts in parallel with the known proteolytic and nucleolytic DNA–protein cross-link repair pathways. To shed light on the possible mechanism of SMC5/6 action, we focused on the small ubiquitin modifier (SUMO) ligation activity of this complex. We showed the SMC5/6 complex-dependent accumulation of SUMO at the crosslinked foci induced by zebularine.

**Next steps:** We will focus further on the role of SUMO in plant DNA damage repair and will characterize other HZE candidates coming from the forward-directed genetic screen. This will help us understand the mechanisms of DNA–protein cross-link repair in plants.

## Introduction

Cellular DNA is constantly exposed to various genotoxic factors that may alter its structure and result in DNA lesions. A common type of DNA damage is DNA–protein cross-links (DPCs), which form when proteins covalently bind to DNA. DPCs are among the most toxic yet least studied lesions that impede DNA-related processes. Indeed, if not repaired, DPCs may lead to mutations, genomic instability, and eventually cell death (Barker et al., 2005). Based on their nature and origin, DPCs can be classified into three main categories: enzymatic, non-enzymatic, and DPC-like traps (Zhang et al., 2020). Enzymatic DPCs occur with proteins that form short-term covalent reaction intermediates as part of their enzymatic cycle (e.g. topoisomerases, DNA methyltransferases). Such DPCs are formed by stabilizing the covalent bond with a specific poison. Non-enzymatic DPCs are caused by the covalent crosslinking of proteins located in the vicinity of DNA. Last, DPC-like trapping occurs when a protein becomes firmly bound to DNA and behaves as a DPC (Stingele et al., 2016, 2017; Klages-Mundt and Li, 2017; Weickert and Stingele, 2022).

Both endogenous and exogenous DPC inducers have been described. Endogenous crosslinkers occur naturally in cells as products of metabolism and include reactive aldehydes such as acetaldehyde and formaldehyde (Nakamura and Nakamura, 2020). Exogenous crosslinkers are induced environmentally, e.g. after exposure to ultraviolet (UV) or ionizing radiation (Kojima and Machida, 2020). Therapeutic crosslinkers represent particular types of exogenous crosslinkers that were identified as potent chemotherapeutic agents. Well-known examples of enzymatic poisons that intercalate at the DNA–protein interface and cause covalent trapping of the target protein to DNA are camptothecin (CPT), etoposide and 5-azacytidine or zebularine, which cross-link TOPOISOMERASE 1 (TOP1; type-3 DPC) (Pommier and Marchand, 2012), TOPOISOMERASE 2 (TOP2; type-4 DPC) (Nitiss, 2009), or DNA METHYLTRANSFERASE 1 (DNMT1/MET1; type-1 DPC) (Maslov et al., 2012; Prochazkova et al., 2022), respectively.

Owing to the structural and chemical diversity of the proteins that can be crosslinked and the DNA contexts in which they occur, DPCs can be challenging lesions for repair. Several DPC repair pathways have been reported (Pouliot et al., 1999; Regairaz et al., 2011; Stingele et al., 2014; Sun, Jenkins, et al., 2020, Sun, Saha, et al. 2020). First, proteolytic cleavage of the protein component of DPCs includes the recently identified metalloproteases Weak suppressor of Smt3 (Wss1) in yeasts and SPARTAN (SPRTN) in animals (Stingele et al., 2016; Vaz et al., 2016). Wss1/SPRTN proteolytic activity has no defined protein specificity but depends on DNA binding. Second, direct enzymatic hydrolysis of the 3' phosphate from DNA and the active tyrosyl residue of class I Topoisomerases was described, catalyzed by Tyrosyl-DNA phosphodiesterase 1 (TDP1) in yeast *Saccharomyces cerevisiae* (Pouliot et al., 1999). Wss1 and TDP1 define parallel genetic pathways for the repair of CPT-induced DPCs in yeast (Stingele and Jentsch, 2015). The Arabidopsis (*Arabidopsis thaliana*) genome contains two *Wss1* homologs, *WSS1A* and *WSS1B* (Enderle et al., 2019). However, only *wss1a* mutant plants were hypersensitive to the DPC-inducing agents camptothecin (CPT) and cisplatin, and no additive phenotype was observed in the *wss1a wss1b* double-mutant. Therefore, WSS1A is currently thought to be the only protease involved in the DPC repair in Arabidopsis. Moreover, *wss1a* plants showed severe growth defects and reduced fertility, probably due to the accumulation of natural DPCs. In contrast to animals, TDP1 only plays a minor role in the repair of TOP1 cross-links in Arabidopsis and may function as a backup pathway to MUS81 and WSS1A-mediated repair (Enderle et al., 2019). Additionally, TDP2 contributes to the repair of TOP2 cross-links in Arabidopsis (Hacker et al., 2022). Last, DPCs can be directly processed by DNA endonucleases. The heterodimeric MMS AND UV SENSITIVE 81 (MUS81) and ESSENTIAL MEIOTIC ENDONUCLEASE 1A (EME1) endonuclease complex acts preferentially on DNA substrates that mimick stalled replication forks, nicked Holliday junctions (HJs), and D-loops (Chen et al., 2001; Doe et al., 2002). In Arabidopsis, MUS81 processes HJs, aberrant replication intermediates, and acts in homologous recombination (HR) (Hartung et al., 2006; Mannuss et al., 2010). Plants lacking MUS81 activity are hypersensitive to CPT and cisplatin, indicating their possible function in processing DPCs next to single-strand breaks or stalled replication forks (Enderle et al., 2019).

The STRUCTURAL MAINTENANCE OF CHROMOSOMES 5/6 (SMC5/6) complex is an evolutionary conserved DNA-stimulated ATP-dependent molecular machine involved in organizing DNA and preserving genome stability. The core SMC5/6 complex is composed of the ring structure of SMC5 and SMC6 heterodimers and several NON-SMC ELEMENT (NSE) subunits (Diaz and Pecinka, 2018; Palecek, 2019). The SUMO-ligase subunit NSE2 is positioned at the SMC5 arm, and SUMOylates HR factors to stimulate DNA damage repair (Varejão et al., 2018; Whalen et al., 2020). SUMO modification of DPCs also facilitates their repair (Schellenberg et al., 2017; Borgermann et al., 2019) but has not been connected to the SMC5/6 complex so far.

The Arabidopsis genome encodes two SMC6 (SMC6A, SMC6B), one SMC5 and six NSE subunits [NSE1–3, NSE4A and NSE4B, ARABIDOPSIS SNI1 ASSOCIATED PROTEIN 1 (ASAP1), and SUPPRESSOR OF NPR1-1, INDUCIBLE 1 (SNI1)]. However, only SMC6B, NSE2, and NSE4A have been firmly associated with DNA damage repair (Watanabe et al., 2009; Liu et al., 2015; Díaz et al., 2019). Our understanding of biological events controlled by the plant SMC5/6 complex and its individual subunits is rather limited. Mutants defective in each subunit are hypersensitive to DNA-damaging treatments, show delayed repair of DNA strand breaks, and accumulate toxic replication intermediates originating during somatic and meiotic HR (Liu et al., 2015; Diaz et al., 2019; Nowicka et al., 2020; Yang et al., 2021), but the exact repair mechanism is unknown.

Recently, we showed that zebularine caused enzymatic DPCs in Arabidopsis by covalently trapping the DNMT1 ortholog MET1 to DNA (Prochazkova et al., 2022). The presence of zebularine-induced DPC is signaled by both ATM and ATR kinases (Liu et al., 2015) and triggered genome instability (Nowicka et al., 2020). Here, we introduce a forward genetic screen aimed at the identification of genes involved in the repair of zebularine-induced DNA damage and present the first mapped complementation group *HYPERSENSITIVE TO ZEBULARINE 1* (*HZE1*, pronounced as “haze”, to refer to the long unclear DNA-damaging effects of zebularine). We mapped the high-effect candidate gene *HZE1* to the *SMC6B* locus. Using several DNA–protein crosslinking agents and constructing higher-order mutants, we show that SMC5/6 repairs DPCs in parallel to known DPC repair pathways. Furthermore, our data suggest that the SUMOylation of MET1-DPCs by the SMC5/6 complex is involved in DPC repair.

## Results

### A forward genetic screening identifies *HZE1* as *SMC6B*

**Figure 1:**
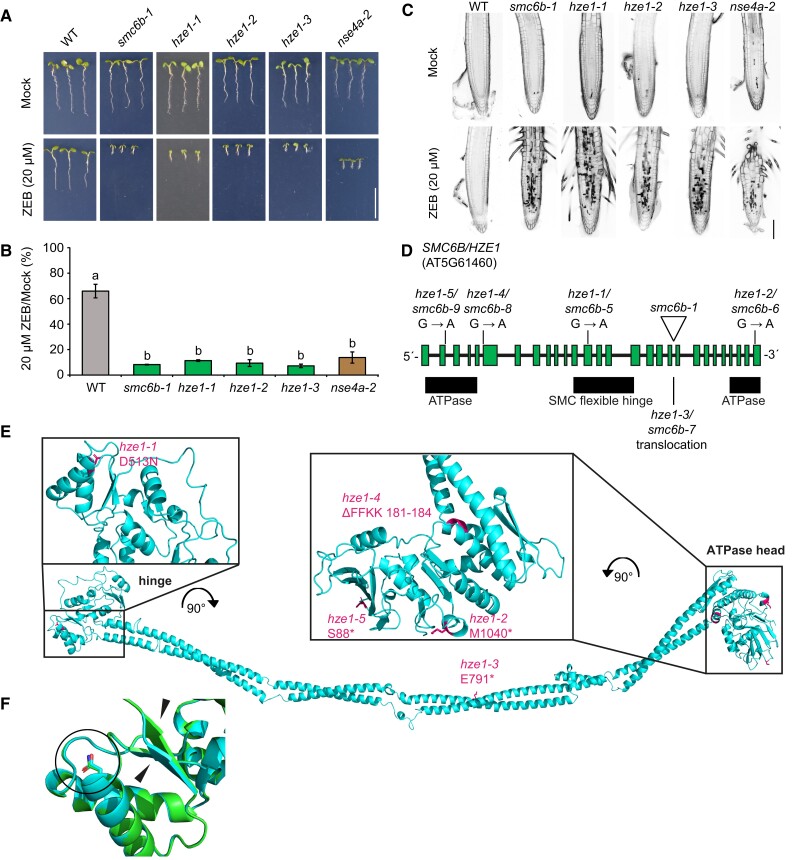
*HYPERSENSITIVE TO ZEBULARINE 1* (*HZE1*) encodes the SMC5/6 complex subunit SMC6B. A, Representative growth phenotypes of seedlings from wild-type (WT), *smc6b-1*, *hze1* alleles and *nse42-2* on 0 (Mock) and 20 µM zebularine (ZEB). Scale bar, 1 cm. B, Relative root length of seedlings in (A) under zebularine/mock conditions (% of ZEB/Mock). Data are means ± SD from three biological replicates, each with a minimum of 14 seedlings. Different lowercase letters indicate significant differences (*P* < 0.05), according to one-way ANOVA followed by Tukey's test. Source data for statistical analyses are available in Supplemental Table 1. The original experiment was split between Figures 1, A–C, and Supplemental Figures 5, A–D. Therefore, these figures show identical images and data for the controls. C, Representative confocal microscopy images of root tips stained with propidium iodide, which indicates dead cells (dark sectors). Five-day-old seedlings were treated with 20 µM ZEB for 24 h prior to analysis. Scale bar, 100 µm. D, Schematic model of the *SMC6B/HZE1* locus (At5g61460) with the positions of individual mutations. E, Detailed position of *hze1-1* to *hze1-5* mutations (magenta) in the AtSMC6B protein structure. The AtSMC6B (UniProt ACC: Q9FIIH) model was built using SWISS-MODEL using *Saccharomyces cerevisiae* SMC6 (PBDID: 7qcd) (Hallett et al., 2022) as template. F, Superposed models of the hinge domain from wild-type SMC6B (azure) and SMC6B in *hze1-1* (green) generated using AlphaFold2. Position of the D513N substitution is marked with black circle. The predicted effect on secondary structure is marked with black asterisks.

For the genetic screen, we mutagenized seeds of the Arabidopsis W35 line (Willing et al., 2016) (hereby referred to as wild-type, WT) with ethyl methanesulfonate (EMS) and screened M2 seedlings for a decrease in root length when grown on half-strength Murashige and Skoog (MS) medium containing 7.5 µM zebularine. We validated the candidate mutant using M_3_ seedling grown in the presence of 20 µM zebularine; we considered the candidates as positive when showing at least a 60% reduction in root length compared to their mock-treated control (for details, see Materials and Methods and Supplemental Figure 1). For reference, the reduction in root length of treated WT seedlings relative to untreated WT was 40%. The first candidate identified in the screen showed an over 90% reduction in root length (9.4% ± 2.7% of mock-treated seedlings), indicating a strong sensitivity to zebularine (Figure 1, A–C, Supplemental Table 1). We named this candidate *hze1-1* for *hypersensitive to zebularine 1*. We performed mapping-by-sequencing (MBS) to identify the causal gene using a pool of ∼100 F_2_ zebularine-sensitive seedlings derived from a backcross to WT (Supplemental Figure 2A). We located the *hze1-1* mutation to the telomere-proximal region on the bottom arm of chromosome 5 (Supplemental Figure 2B). We analyzed this region for moderate to high-effect mutations within protein-coding regions and identified a G-to-A transition in *SMC6B* at 3,627 bp downstream from the ATG (Figure 1D, Supplemental Figure 2C), resulting in a D513N substitution. Notably, the putative causal mutation in *hze1-1* was located within the SMC6B hinge domain (Figure 1, E and F) in the highly conserved α-helix of subdomain I (Alt et al., 2017) that is responsible for proper folding (Figure 1F). Homology modeling using the budding yeast SMC6B crystal structure (Hallett et al., 2022) as template revealed that the D513N substitution likely causes a subtle change in charge of the SMC6B hinge domain, which may affect proper folding of the hinge domain (Figure 1F). We validated *SMC6B* as the causal gene by analyzing sensitivity to zebularine in F_1_ seedlings from a cross between *hze1-1* and *smc6b-1,* which confirmed that *HZE1* is allelic to *SMC6B* (Supplemental Figure 3).

To assess whether other mutant alleles in our collection affect *SMC6B*, we analyzed the phenotypes of the remaining selected *hze* mutants on zebularine and found four additional zebularine hypersensitive but otherwise phenotypically WT-like candidates. We performed complementation crosses between these candidates and *smc6b-1*, followed by zebularine sensitivity assays, which suggested that they are all allelic (Supplemental Figure 3). Consequently, these candidates were named *hze1-2* to *hze1-5* (Figure 1, A–D, Supplemental Figure 3, Supplemental Table 1). We sequenced their *SMC6B* cDNA by Sanger sequencing and modeled the effect of the identified substitutions using the *in silico* predicted SMC6B structure (Figure 1, D and E). In *hze1-2*, we detected a G-to-A transition 7,233 bp downstream of the *SMC6B* ATG, which overlapped with a splicing donor/acceptor site. The *hze1-2* mutation resulted in alternative splicing of exon 28 that generated a 10-bp deletion, introducing a premature stop codon in the sequence encoding the Walker B motif of the ATPase head domain of SMC6B (Figure 1, D and E). In *hze1-3*, we initially did not find any mutations, but we failed to amplify one genomic region using primer pairs validated on WT genomic DNA. We hypothesized that this region might be rearranged and therefore used inverse PCR for isolation. Indeed, the sequencing of inverse PCR products suggested a reciprocal translocation between chromosomes 5 and 4 with a breakpoint 5,472 bp downstream of the *SMC6B* ATG and its fusion with a fragment of *NEXT TO BRCA1 GENE 1* (*NBR1*) (Figure 1, D and E, Supplemental Figure 4). We confirmed the translocation by a standard PCR assay with individual primers positioned in *SMC6B* and *NBR1*, respectively (Supplemental Figure 4). The *hze1-4* mutant carried a G-to-A transition 1,332 bp downstream of the *SMC6B* ATG (Figure 1, D and E, Supplemental Figure 3), which overlapped with a splicing donor/acceptor site and resulted in the deletion of four amino acids (181–184, ΔFFFK) in the DNA-binding motif of the ATPase head domain SMC6B (Yu et al., 2022). The *hze1-5* mutant had a G-to-A transition 264 bp downstream of the *SMC6B* ATG (Figure 1, D and E, Supplemental Figure 3), which overlapped with a splicing donor/acceptor site and caused the retention of the 3rd intron in the final transcript. This retained intron added nine amino acids and a premature stop codon in the ATPase head domain (after amino acid 97). To exclude the possibility that sensitivity to zebularine was due to an SMC6B function independent from the SMC5/6 complex, we also tested *nse4a-2,* which carries a mutation in the kleisin subunit of the complex (Díaz et al., 2019) and confirmed its strong sensitivity to 20 µM zebularine (Figures 1, A–C). Analyses of root cell viability using propidium iodide (PI) staining revealed an increased number of dead cells in the root meristematic zone of *hze1* and control *smc6b-1* and *nse4a-2* seedlings (Figure 1C). The *nse4a-2* seedlings showed fewer dead cells, most likely because this is not a null mutant allele (Díaz et al., 2019).

In conclusion, we identified five new EMS-induced *SMC6B* mutant alleles, *hze1-1* to *hze1-5* (corresponding to *smc6b-5* to *smc6b-9* alleles), and showed that the SMC5/6 complex participates in the repair of zebularine-induced type 1 DPCs.

### The SMC5/6 complex is also involved in the repair of TOP1 and TOP2 DPCs

**Figure 2:**
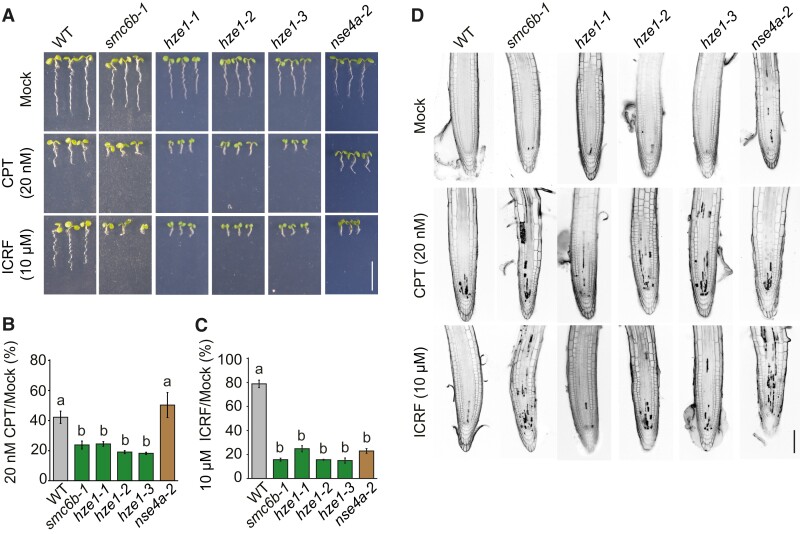
The SMC5/6 complex is required for the repair of Type 3 and Type 4 DNA–protein cross-links (DPCs). (A), Representative growth phenotype of wild-type (WT) and mutant seedlings on medium without DPC inducers (Mock) or containing 20 nM camptothecin (CPT) or 10 µM ICRF-187 (ICRF). Scale bar, 1 cm. B, C, Relative root length of WT and mutant seedlings grown in the presence of 20 nM CPT (B) or 10 µM ICRF (C). Data are means ± SD from three biological replicates, each with at least 20 seedlings. Different letters indicate significant differences (*P* < 0.05) according to one-way ANOVA followed by Tukey's test. Source data for statistical analyses are available in Supplemental Table 2. The original experiment was split between Figure 2, A–C and Supplemental Figures 5, A–D. Therefore, these figures show identical images and data for the controls. D, Representative confocal microscopy images of root apices stained with propidium iodide. Five-day-old seedlings were treated for 24 h with 20 nM PT or 10 µM ICRF prior to analysis. Dark sectors within the roots indicate dead cells. Scale bar, 100 µm.

The severity of *hze1* hypersensitivity to zebularine raised the question as to whether the SMC5/6 complex might also be involved in the repair of other types of DPCs. Accordingly, we analyzed root length in response to 20 nM CPT treatment. CPT cross-links TOP1 and induces type-3 DPCs (Hacker et al., 2020). Root length in CPT-treated WT seedlings was 41.9% ± 0.7% that of untreated control seedlings, while *smc6b-1* mutant seedlings displayed a significantly stronger reduction in root length, reaching only 23.4% ± 2.6 of the original root length (one-way ANOVA and Tukey's HSD post hoc test, *P* < 0.05; Figure 2, A and C; Supplemental Table 2). In addition, CPT treatment shortened the meristematic zone and increased cell death in the roots of *smc6b-1* seedlings (Figure 2D). We observed a similar sensitivity in all tested *hze1* alleles (Figure 2, A and B, 2D). Although *nse4a-2* seedlings showed a relatively strong reduction in root length (50.3% ± 8.3%), this effect was not significantly different from that of WT seedlings (*P* = 0.169), consistent with the classification of the *nse4a-2* mutant as a hypomorphic allele (Diaz et al., 2019). In agreement with published data, we observed sensitivity to CPT for *mus81-1* and *wss1a-1* seedlings, but not for *tdp1-3* or *tdp2-5* (Supplemental Figure 5, B and, 5E) (Enderle et al., 2019; Hacker et al., 2022).

Second, we analyzed the role of the SMC5/6 complex in the repair of Type 4 DPCs, typically associated with the crosslinking of TOP2 to its cleavage sites. This type of DPC is also caused by the TOP2 poison etoposide, which inhibits the religation of cleaved DNA segments, resulting in TOP2 binding to the cleaved DNA ends (reviewed in Nitiss, 2009). Because etoposide shortens root length only at very high concentrations (Hacker et al., 2022), we screened several other TOP2 poisons and inhibitors used in mammalian research and observed a strong effect after treatment with bisdioxopiperazine dexrazoxane (ICRF-187) (Figure 2, B–D, Supplemental Table 2). Seedlings from *smc6b-1*, *nse4a-2,* and all tested *hze1* alleles (−*1* to −*3*) showed a massive root length reduction to less than 25% of mock-treated controls, while the root length in WT seedlings was only weakly affected in response to 10 µM ICRF-187, with a root length of 81.0% ± 1.7% relative to the mock treatment. In all cases with significantly reduced root length, we also detected more dead cells in the root meristems of *smc6b-1*, multiple alleles of *hze1* and *nse4a-2* seedlings (Figure 2D). We also observed hypersensitivity to ICRF-187 in *wss1a-1,* which was in agreement with the known function of WSS1A in the repair of TOP2 cross-links (Hacker et al., 2022) (Supplemental Figure 5, C–E, Supplemental Table 3). ICRF-187 is a highly specific TOP2 inhibitor that links the interface between two ATPase protomers of TOP2 (Classen et al., 2003; Nitiss, 2009; Lee et al., 2017, 2022). To test the role of the SMC5/6 complex in the repair of Type 2 cross-links, we tested several chemicals known to induce Poly (ADP-ribose) polymerase 1 (PARP1) DPCs in mammalian cells (Waldman and Waldman, 1990; Bernges and Zeller, 1996; Menear et al., 2008). However, there were no visible differences between WT and *smc6b-1* (Supplemental Figure 6), preventing us from evaluating the repair of Type 2 cross-links. Collectively, these findings provide strong evidence that the SMC5/6 complex is a critical component in the repair of different types of DPCs and establish ICRF-187 as a new drug for plant DPC repair research.

### SMC6B, MUS81, and WSS1A function non-redundantly during the repair of endogenous DNA damage

**Figure 3:**
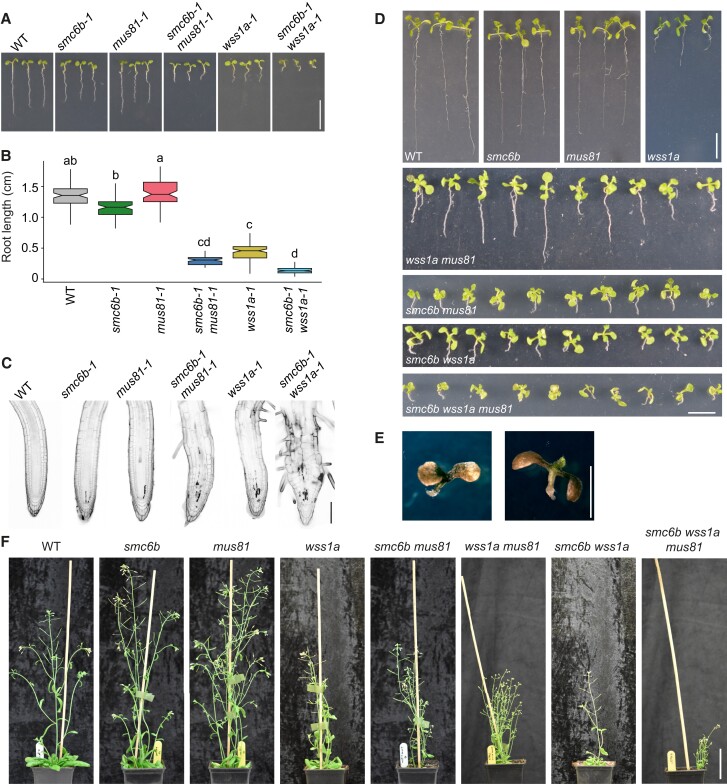
Phenotypic analysis of *smc6b-1*, *mus81-1*, *wss1a-1* and their higher-order mutants under normal conditions. A, Representative growth phenotype of wild-type, *smc6b-1*, *mus81-1*, *wss1a-1* and double mutants with *smc6b-1*. Scale bar, 1 cm. (B), Quantification of root length from A. At least 20 roots per genotype were analyzed in each of three biological replicates. The lower and upper hinges of the boxplots correspond to the first and third quartiles of the data, the black lines within the boxes indicates the median. Whiskers mark 10% and 90% intervals. Different letters indicate significant differences (*P* < 0.05) according to one-way ANOVA followed by Tukey's test. Source data for statistical analyses in B are available in Supplemental Table 4A. (C), Representative confocal microscopy images of root tips stained with propidium iodide. Seedlings were grown for five days on control medium prior to analysis. Dark sectors indicate dead cells. Scale bar, 100 µm. (D), Representative phenotypes of two-week-old WT, single mutants, double mutants, and *smc6b-1 mus81-1 wss1a-1* triple mutant seedlings grown on half-strength MS medium. Scale bar, 1 cm. (E), Detailed photograph of three-week-old *smc6b-1 mus81-1 wss1a-1* triple mutant seedlings with severe phenotype grown on half-strength MS medium. Scale bar, 1 cm. (F), Representative phenotypes of six-week-old plants grown on soil. Scale bar, 70 mm.

The endonuclease MUS81 and the protease WSS1A are required for DPC repair in Arabidopsis (Enderle et al., 2019). To uncover a possible genetic interaction between the SMC5/6 complex and these factors, we generated *mus81-1 smc6b-1* and *wss1a-1 smc6b-1* double-mutant plants and analyzed them under mock conditions with spontaneously occurring DNA damage (Figure 3, A and B, Supplemental Table 4). The root length of *smc6b-1* (1.18 cm ± 0.11) and *mus81-1* (1.41 cm ± 0.11) seedlings was comparable to that of WT seedlings (1.34 cm ± 0.03), while the roots of *wss1a-1* (0.44 cm ± 0.05 cm) were significantly shorter (one-way ANOVA and Tukey's HSD post hoc test, *P* < 0.05, note: the same test was used throughout this section). The *smc6b-1 mus81-1* double-mutant grown under mock conditions showed a significant 75% reduction in root length (0.31 cm ± 0.06 cm) relative to mock-treated WT (Figure 3A, B). Furthermore, we observed more dead cells in the root meristems of *smc6b-1 mus81-1* seedlings compared to WT and the respective single mutants (Figure 3C). This increased number of dead cells was accompanied with modest changes in root morphology (Figure 3C). The roots of *smc6b-1 wss1a-1* seedlings (0.14 cm ± 0.01 cm) showed a drastic 90% length reduction relative to WT (Figure 3, A and B), and their anatomy was compromised with irregularly positioned and sized cells, a minimal meristematic zone, and root hairs close to the root tip (Figure 3C). Although the total number of dead cells in this double-mutant appeared similar to that of *wss1a-1* seedlings (Figure 3C), we speculate that this may reflect a bias caused by generally fewer cells in the root meristem and transition zones. Adult *smc6b-1* and *mus81-1* single mutant plants were indistinguishable from WT, but the *smc6b-1 mus81-1* double-mutant showed severe growth defects, including tiny rosettes and a shorter stem height by about 40% (Figure 3, D and F). Similarly, *smc6b-1 wss1a-1* double-mutant plants also had a smaller rosette size and were generally shorter compared to WT and single mutant controls (Figure 3, D and F). Altogether, these results indicate that the SMC5/6 complex functions in pathways parallel to MUS81 and/or WSS1A in the repair of spontaneously occurring DNA damage.

To explore whether the SMC5/6 complex contributes to both the MUS81 and WSS1A pathways or whether it represents an independent yet unidentified pathway, we generated the *smc6b-1 mus81-1 wss1a-1* triple mutant by crossing the above described homozygous double-mutant plants. We grew three independent *smc6b-1 mus81-1/MUS81 wss1a-1/WSS1a* F1 plants and expected 25% triple homozygous offspring upon selfing. However, we obtained only 1% to 4% seedlings with the triple mutant genotype (2 of 200, 4 of 200, and 8 of 200, in three independent replicates), indicating an additive effect on plant lethality. Several triple homozygous mutant plants were at least partially fertile, allowing us to analyze the phenotype of the progeny closer (Figure 3, D and F). We selected the ten best-looking plants for each double and triple mutant combinations from equally sized populations (Figure 3D). The *smc6b-1 mus81-1 wss1a-1* plants showed stunted growth compared with the respective double-mutant plants, never developed proper roots or shoots, and were smaller compared with the double mutants. Moreover, they were often dark-colored after prolonged cultivation on MS medium (Figure 3F).

Based on these findings, we conclude that the SMC5/6 complex, MUS81, and WSS1A function in at least partially unique pathways during the repair of spontaneously occurring DNA damage.

### SMC5/6, MUS81, and WSS1A act additively during the repair of zebularine-induced DPCs

**Figure 4:**
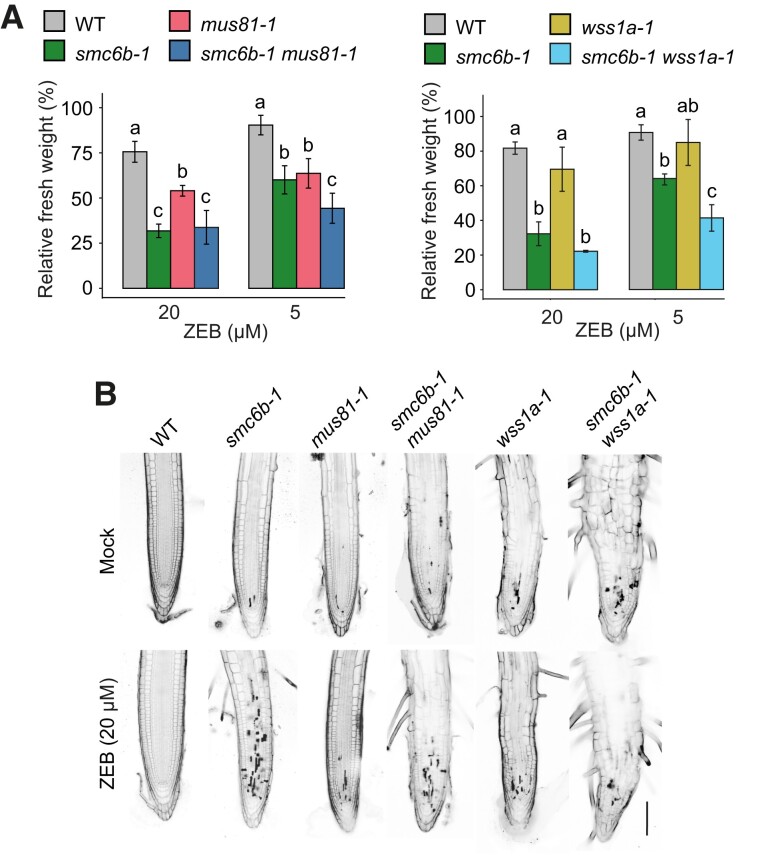
Sensitivity of *smc6b-1 mus81-1* and *smc6b-1 wss1a-1* plants to zebularine. (A), Fresh weight of plants treated with 5 µM or 20 µM zebularine (ZEB). The sensitivity of double-mutant plants was compared with the respective single mutants and WT plants relative to the mock-treated plants of the same genotype. Data are means ± SD of three biological replicates. Different letters indicate significant differences (*P* < 0.05) according to one-way ANOVA followed by Tukey's test. Source data for A are available in **Supplemental Table 4**. (B), Representative confocal microscopy images of root tips stained with propidium iodide. Scale bar, 100 µm.

To uncover whether the SMC5/6 works together with MUS81 and WSS1A in the repair of type-1 DPCs, we tested the zebularine sensitivity of *mus81-1 smc6b-1* and *wss1a-1 smc6b-1* double-mutant plants, which revealed a dose-dependent phenotype (Figure 4A, Supplemental Table 5). The double mutants showed a significant additive hypersensitivity, compared to both single mutants, in response to a low zebularine concentration of 5 µM (one-way ANOVA and Tukey's HSD post hoc test, *P* < 0.05). On the contrary, a higher zebularine concentration of 20 µM fully inhibited *smc6b-1* growth, and we observed no further enhancement of sensitivity in the double mutants. The PI staining of 20 µM zebularine-treated *smc6b-1 mus81-1* seedlings showed moderately altered root anatomy with uneven cell files and a similar number of dead cells as in the root meristematic zone of *smc6b-1* seedlings (Figure 4B). That differences are best observed at the lower zebularine concentration for the fresh weight assay, and at the higher concentration in the cell death assays reflects the different durations of these experiments.

WSS1/SPRTN proteins have a unique role in DPC repair (Stingele et al., 2016; Reinking et al., 2020). Therefore, testing the sensitivity of *smc6b-1 wss1a-1* to zebularine allowed us to unambiguously test the role of the SMC5/6 complex in DPC repair (Figure 4A, Supplemental Table 5). Again, a high-dose zebularine treatment caused the greatest sensitivity in *smc6b-1*, and we did not observe a further increase in the sensitivity of the double mutants. For the 5 µM zebularine dose, we measured a statistically significant additive effect (one-way ANOVA and Tukey's HSD post hoc test, *P* < 0.05) in the *smc6b-1 wss1a-1* seedlings compared with the WT and respective single mutants. Due to the severely affected root anatomy and a high number of dead cells under mock conditions, we could not precisely estimate the effect of zebularine on the number of dead cells within the root meristematic zone of *smc6b-1 wss1a-1* seedlings (Figure 4B). In general, the roots were short and appeared very thick with minute meristematic zones. Because of the severe developmental phenotype of the *smc6b-1 mus81-1 wss1a-1* triple mutants, we were not able to analyze their response to zebularine.

In conclusion, our findings indicate that the SMC5/6 complex acts in a parallel pathway to MUS81 for the repair of zebularine-induced DPCs. Moreover, we provide solid genetic evidence that the SMC5/6 complex is specifically involved in DPC repair and functions in the pathway(s) parallel to WSS1A protease.

### SUMOylation targets MET1 cross-links in an SMC5/6-dependent manner

**Figure 5:**
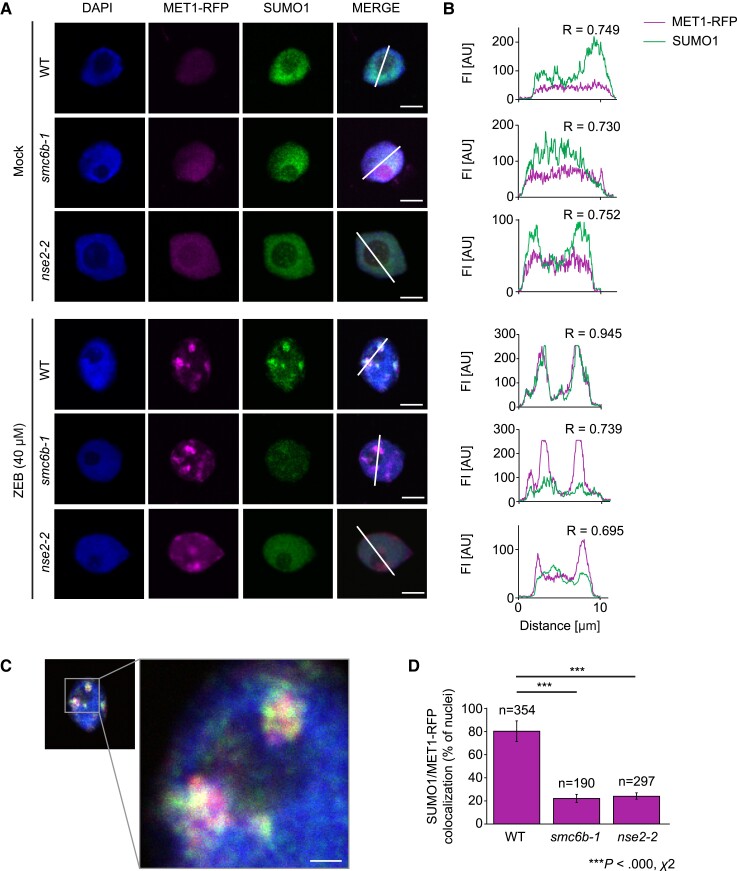
SMC5/6-dependent SUMOylation of zebularine-induced MET1 cross-links. (A), Immunolabeling of mock- and zebularine-treated WT, *smc6b-1*, and *nse2-2* root nuclei stained with SUMO1 antibody. MET1-RFP signals were observed directly, and nuclei were counterstained with DAPI. The white lines in Merge indicate intersects for fluorescence intensity measurements shown in (B). Scale bar, 5 µm. (B), Fluorescence intensity (FI) plots based on the white lines indicated intersects in (A). The y-axis shows Fl intensity in arbitrary units (AU) for MET1-RFP and SUMO1 signals. R indicates Pearson's correlation coefficient assessment of colocalization (1, full colocalization). (C), Detailed image of MET1-RFP colocalization with SUMO1 (from A) in WT nuclei after zebularine treatment. Scale bar, 1 µm. (D), Percentage of nuclei with MET1-RFP foci colocalizing with SUMO1 in WT, *smc6b-1* and *nse2-2* root nuclei after zebularine treatment. Data are means ± SD from three biological replicates. Statistical significance was tested with chi-square test (*smc6b-1* x^2^ (2, N = 544) = 196.6331, *P* = 0.000, *nse2-2* (x^2^ (2, N = 651) = 231.7348, *P* = 0.000). n, total number of nuclei evaluated per genotype. Source data for the analyses are available in Supplemental Table 6.

In animals, proteins covalently trapped to DNA are targeted by SUMOylation (Borgermann et al., 2019; Liu et al., 2021; Ruggiano et al., 2021). The SMC5/6 complex contains the evolutionary conserved E3 SUMO-ligase subunit NSE2 (Varejão et al., 2018). Therefore, we wondered whether SMC6B might link the repair of zebularine-induced DPCs with the SUMOylation activity of the SMC5/6 complex. We previously showed that zebularine induces cytologically detectable DPC arrays at *45S* rDNA in Arabidopsis by crosslinking a large number of fluorescently-tagged MET1 (Prochazkova et al., 2022). To establish whether zebularine-induced MET1-DPCs are targeted for SUMOylation, we analyzed SUMO enrichment at MET1-RFP (red fluorescent protein) foci after zebularine treatment (Figure 5A). To this end, we performed immunolabeling with antibodies specific to SUMO1 or SUMO3 on MET1-RFP-positive nuclei isolated by flow sorting from mock- and zebularine-treated wild-type plants as described (Prochazkova et al., 2022). We observed no immunostaining of foci with antibodies against SUMO3 (Supplemental Figure 7). By contrast, SUMO1 showed dispersed signals under mock conditions but largely colocalized with MET1-RFP foci after a 40-µM zebularine treatment (Figure 5, A and B). To test whether SMC5/6 is responsible for the zebularine-induced deposition of SUMO1 on MET1-DPCs, we repeated the immunolabeling with the anti-SUMO1 antibody on MET1-RFP-positive nuclei from *smc6b-1* seedlings. Indeed, most zebularine-stimulated SUMO1 localization of MET1-RFP foci was effectively abolished in the *smc6b-1* background (Figure 5, A and B, D). Wild-type plants showed 81% ± 9% colocalization between MET1-RFP and SUMO1 signals upon zebularine treatment, while *smc6b-1* reached only 22% ± 4% (Figure 5D). To unequivocally support a role for the SMC5/6 complex in SUMOylation at MET1-RFP foci, we repeated the experiment with the *nse2-2 MET1-RFP* line. As with *smc6b-1*, about 24% ± 3% of *nse2-2* nuclei showed SUMO1 colocalization with MET1-RFP (Figure 5, A and B, D).

This finding shows that the SMC5/6 complex adds SUMO1 to crosslinked MET1-RFP upon zebularine treatment, thus highlighting the importance of the SMC5/6 complex in SUMOylation of DPCs. The persistence of SUMO1 at around 20% of MET1-RFP foci also suggests a role for another E3 SUMO-ligase in labeling a subset of DPCs.

## Discussion

Here, we describe the SMC5/6 complex as an important component involved in the repair of DNA–protein cross-links, possibly through its E3 SUMO-ligase activity. DPCs are highly toxic DNA adducts that represent a major threat to the maintenance of genome integrity (reviewed in Weickert and Stingele, 2022). DPCs, such as TOP1 cross-links, are formed during normal plant metabolism but are rapidly removed through a number of repair pathways involved in their elimination. Unlike the repair of other types of DNA damage, detoxification of DPCs has been studied in depth only recently (Stingele and Jentsch, 2015; Vaz et al., 2016; Enderle et al., 2019; Larsen et al., 2019; Reinking et al., 2020; Liu et al., 2021). In plants, three major DPC repair pathways have been described to date: the nucleolytic pathway, which is hallmarked by the structure-specific endonuclease MUS81 (Enderle et al., 2019); the DPC-specific proteolytic pathway that depends on the WSS1/Spartan metalloproteases (Stingele et al., 2016; Enderle et al., 2019); and the direct hydrolytic pathway represented by TDP1 and TDP2 (Enderle et al., 2019; Tsuda et al., 2020). We show that the SMC5/6 complex represents an independent or overarching DPC repair pathway.

The function of the SMC5/6 complex is traditionally associated with DNA damage repair and maintenance of genome stability. Using a forward genetic screen, we identified five loss-of-function mutant alleles in *HYPERSENSITIVE TO ZEBULARINE 1* (*HZE1*), a putative key player involved in the repair of zebularine-induced DPCs based on the strong sensitivity of its mutants. The *HZE1* complementation group was allelic to *SMC6B*, a gene encoding the core subunit of the SMC5/6 complex. The *hze1-2* mutant harbors a mutation in the ATPase domain and most likely leads to a catalytically dead SMC6B. The *hze1-3* allele carries a large translocation in the 3' end of the gene, effectively breaking the gene into two fragments. The *hze1-4* allele lacks four amino acids in the ATPase head domain necessary for interaction with DNA (Yu et al., 2022). Finally, the *hze1-1* allele represents a unique mutation within subdomain I of the hinge region (Alt et al., 2017). Alignment of this region from SMC6 homologs from different organisms revealed that it is highly conserved among plant, fungal and animal species (Supplemental Figure 8). It is likely that the Asp-to-Asn mutation in *hze1-1* results in aberrant chemical properties of the hinge domain, thus affecting its structure and/or flexibility. We selected the HZE mutants based on their sensitivity to zebularine, which is a cytidine analog incorporated into DNA during replication and enables irreversible trapping of the DNA methyltransferase MET1 in plants (Prochazkova et al., 2022). The exact repair pathway of zebularine MET1-DPCs is not known but sensitivity studies indicate that the HR pathway is involved (Liu et al., 2015; Nowicka et al., 2020). The isolation of *SMC6B* in our mutant screen is also in agreement with the role of the SMC5/6 complex, as SMC6B is required for efficient DNA damage repair by HR (Mengiste et al., 1999; Potts et al., 2006; Watanabe et al., 2009). The sensitivity to zebularine of a partial loss-of-function mutant in the kleisin subunit NSE4A indicates that the SMC5/6 complex is involved in the DPC repair as a whole (Díaz et al., 2019).

Here we show that the SMC5/6 complex is a universal player in DPC repair, as it was not only involved in the repair of MET1 crosslinked to DNA by zebularine (representing type-1 DPCs) but also in the repair of crosslinked TOP1 (Type 3) and TOP2 (Type 4). Topoisomerases are enzymes that introduce transient DNA breaks to relax supercoiled or intertwined DNA, thus allowing replication- and transcription-associated complexes to proceed and sister chromatids to separate. In search of effective TOP2 inhibitors in plants, we tested several compounds used for animal research and identified ICRF-187 as a new highly potent crosslinker. The most substantial effects were when the drug was applied at early stages of seedling development, possibly concomitant with the large number of replicating nuclei. ICRF-187 cross-links TOP2 in the ATP-associated state around double-stranded DNA (dsDNA), hence creating a cross-link on DNA that is not associated with DNA strand break (Classen et al., 2003; Lee et al., 2022). By contrast, cytidine analogs have a very distinctive mode of action. Drugs like 5-azacytidine are incorporated into DNA and act as a pseudosubstrate for DNA methyltransferases (DNMTs), resulting in the covalent trapping of the enzyme without the primary presence of single-stranded breaks (SSBs) or double-stranded breaks (DSBs). Repairing such cross-links may lead to DSBs (Maslov et al., 2012). We did not observe increased sensitivity of *tdp1-3* or *tdp2-5* seedlings to zebularine-induced DPCs. This result is in agreement with the fact that zebularine induces type-1 DPCs (Hacker et al., 2020) that lack the phosphodiester bond, a common substrate for TDP1 and TDP2. Therefore, the role of the SMC5/6 complex appears more general than that of other DPC repair factors and likely DPC type-independent.

**Figure 6:**
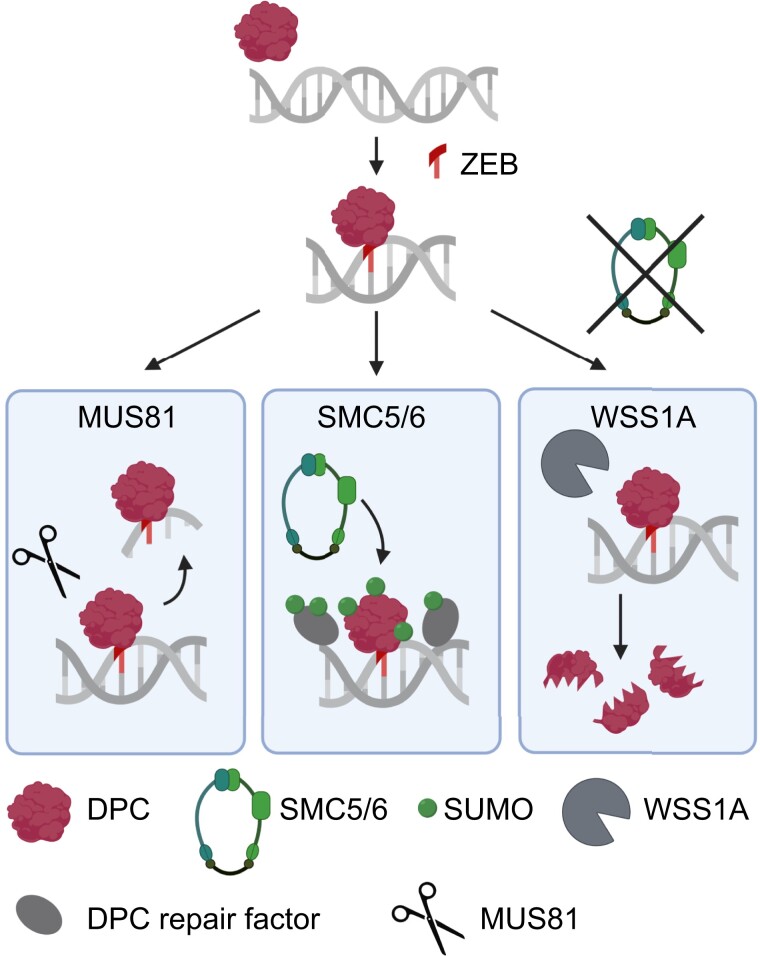
Working model of zebularine-induced DPC repair. The endonuclease MUS81 cleaves DNA surrounding the cross-link. The SMC5/6 complex deposits SUMO residues on the MET1-DPC or adjacent repair proteins to stimulate repair. Without the SMC5/6 complex, the protease WSS1A proteolytically degrades the protein crosslinked by zebularine.

It is tempting to speculate how the SMC5/6 complex is involved in DPC repair (Figure 6). It has been shown that each of the two SMC6 homologs in Arabidopsis is required for the efficient repair of DNA breakage via intermolecular HR in somatic cells (Watanabe et al., 2009). Alignment of sister chromatids is enhanced transiently after X-ray irradiation (and mitomycin C treatment) in WT nuclei. In the SMC5/6 complex mutants, the X-ray–mediated increase in sister chromatid alignment is much lower and delayed than in WT. Therefore, we hypothesize that the function of the SMC5/6 complex might be required for the use of the sister chromatid as a template for repair. This mode of action might not only be restricted to the repair of replicative DSBs by HR but also by post-replicative DPC repair (Liu et al., 2021) in which a template-switching mechanism using the sister chromatid might be involved (Torres-Ramos et al., 2002; Chen et al., 2008).

Interestingly, protein degradation by the proteasome in the replication-independent pathway depends on the prior SUMOylation of the respective proteins (Liu et al., 2021). Conjugation of SUMO has previously been described for several naturally occurring and chemically-induced DPCs, including TOP1, TOP2, and DNMT1 in animals and yeast (Schellenberg et al., 2017; Borgermann et al., 2019; Liu et al., 2021; Serbyn et al., 2021). The E3 SUMO-ligase activity of the SMC5/6 complex might mark crosslinked proteins for degradation (Figure 6) and/or for conjugation with other factors promoting the repair. Hence, SUMOylation via the SMC5/6 complex might be a mechanism integrating and orchestrating various DPC repair pathways in plants. Interestingly, the SUMOylation activity of the SMC5/6 complex is unique among all SMC complexes and canonical DNA damage repair factors. Arabidopsis genome encodes eight SUMO proteins, and four of the encoding genes are expressed (*SUMO1, 2, 3, 5*) (Hammoudi et al., 2016). We discovered here that SUMO1, but not SUMO3, is involved in DPC modification and that this is largely SMC5/6 complex-dependent.

In summary, we identified SMC6B from our forward genetic screen for factors contributing to the repair of zebularine-induced DNA–protein cross-links. SMC6B is a core subunit of the SMC5/6 complex that functions in several DPC repair pathways. We propose that SUMOylation mediated by this complex plays an important role in DPC repair. Further screening and identification of their candidates is in progress and provides a high potential to identify and characterize additional DPC repair factors in Arabidopsis.

## Materials and methods

### Plant materials

Arabidopsis (*Arabidopsis thaliana*) wild-type and mutants in the Col-0 background (unless stated otherwise) were used in this study: *smc6b-1* (SALK_123114C), *nse4a-2* (GK-768H08), *nse2-2* (SAIL_77_G06), *mus81-1* [GABI_113F11, (Hartung et al., 2006)], *wss1a-1* [CRISPR/Cas9 line, (Enderle et al., 2019)], *tdp1-3* (CRISPR/Cas9 line with a 1 bp insertion in exon 1 of *TDP1*, (Enderle et al., 2019)), *tdp2-5* (CRISPR/Cas9 line with a 5 bp deletion in exon 1 of *TDP2*, (Hacker et al., 2022)). The double mutants were generated by crossing homozygous single mutants and identification in the F2 generation by PCR-based genotyping. Plants homozygous for the *wss1a-1* mutation were identified by Sanger sequencing of PCR products spanning the mutated site. The primers used for genotyping are listed in Supplemental Table 7. Plants were cultivated in an air-conditioned phytochamber under a long-day photoperiod (16 h light, 150 µmol m^–2^ s^−1^, 21°C, 8 h dark, 19°C; lights provided by fluorescent tubes MASTER TL-D 18W/840, Philips). For the drug sensitivity assays, seeds were surface-sterilized using 8% (w/v) sodium hypochlorite solution for 6 min, followed by three washes in sterile H_2_O, stratification for 2 days at 4°C in the dark. Seeds were evenly distributed on plates containing half-strength MS medium with 0.6% (w/v) agar and with or without the addition of DNA–protein crosslinking chemicals, depending on the experimental setup.

### Accession numbers

Sequencing data of the mapping populations were deposited at the NCBI Sequence Read Archive under accession number PRJNA730368. Sequence data of the genes used in this article can be found at TAIR under the following accession numbers: *SMC6B* (At5g61460), *NSE4A* (At1g51130), *NSE2* (At3g15150), *MUS81* (At4g30870), *TDP1* (At5g15170), *TDP2* (At1g11800), *WSS1A* (At1g55915).

### *DCPR* forward-directed genetic screen setup, candidate MBS, and inverse PCR

For the HZE genetic screen (Supplemental Figure 1), an ethyl methanesulfonate (EMS)-mutagenized population was used in the W35 reporter line (Col-0 carrying *ProUVR2:UVR2-LUCIFERASE*) background (Willing et al., 2016). The reporter line behaves as wild-type, and *UVR2* expression was not monitored in this study. About 10,000 seeds were soaked in 0.1% (w/v) KCl and shaken at 4°C for 8 h; seeds were then washed in distilled water and incubated in 0.2% (v/v) EMS solution at room temperature for 12 h to induce mutations. Afterwards, seeds were washed 2 × 5 min with 100 mM sodium thiosulfate and 3 × 5 min with water. Finally, the seeds were resuspended in 0.1% (w/v) agarose and spread onto soil surface at a density of ∼100 seeds per 18 × 14 cm tray. All M1 plants were grown until maturity; seeds of all plants from one tray were collected together, resulting in 100 M2 seed batches. Approximately 1,500 seeds per M2 batch were surface-sterilized with 8% (w/v) sodium hypochlorite for 6 min, followed by three washes with sterile water, resuspension in 0.1% (w/v) agarose, and 1,600 seeds were evenly sown onto plates filled with half-strength MS medium containing 20 μM zebularine using a pipette with a sterile cut plastic tip. Each plate included the zebularine-sensitive control *smc6b-1* and resistant wild-type controls. Seedlings were grown in a phytochamber under long-day conditions for 10 days. Afterwards, the plates were visually inspected, and primary candidates with short roots were transferred to soil and grown until maturity, and their M3 seeds were collected. Each primary M2 candidate was further analyzed by phenotyping the M3 generation on half-strength MS medium without or with 20 μM zebularine (∼30 seedlings per experimental point). Based on the phenotype, each candidate was classified into one of the following categories: (i) developmental mutants with short roots on both control and zebularine-containing media; (ii) false positive with less than a 60% reduction in root length on control medium compared to zebularine-containing medium; (iii) true candidates with at least a 60% reduction in root length on control medium compared to zebularine-containing medium. Only Type (iii) candidates were considered for further work.

The candidates selected for mapping were backcrossed to the non-mutagenized wild-type Col-0, and the resulting F2 population was screened on half-strength MS medium containing 20 μM zebularine. Segregation of the zebularine-sensitive phenotype was assessed, which typically matched the expected segregation pattern for a single nuclear recessive locus. About 75 to 150 zebularine-sensitive seedlings were collected, pooled, and their genomic DNA was isolated using a NucleoSpin Plant II kit (Macherey-Nagel). Genomic DNA was sent for sequencing (Novogene LTD, Cambridge, UK), as paired-end 150-bp reads on a Novaseq platform to approximately 50 × coverage. Sequencing data were analyzed using bioinformatics tools available at the public platform usegalaxy.org as described (Prochazkova et al., 2022). The clean reads were mapped to the *Arabidopsis thaliana* reference genome (TAIR10) with bowtie2 using default settings (Langmead et al., 2009; Langmead and Salzberg, 2012). Read sorting, SNP calling, and filtering were performed using tools from the MiModD tool set (http://wbg.wormbook.org/2014/03/23/mimodd-mutation-identification-from-wholegenome-sequencing-data-on-desktop-pcs/) and annotated with the snpEff tool (Cingolani et al., 2012). Sequencing data of the mapping populations were deposited at the NCBI Sequence Read Archive under accession number PRJNA730368. Mapping information of respective candidates was uploaded to the UCSC Genome Browser with the following IDs: http://genome-euro.ucsc.edu/s/KlaProche/candidate%208%2D13%20a.k.a.%20dpcr1.

The *SMC6B* locus was sequenced in the candidates identified as *smc6b* mutants via complementation crosses with *smc6b-1*. Total RNA extraction and first-strand cDNA synthesis were performed as described previously (Nowicka et al., 2020). The *SMC6B* transcript was divided into six regions, amplified with specific primers (Supplemental Table 1) and PCR products were sequenced by Sanger sequencing. To identify the putative rearranged region in *hze1-3*, inverse PCR was performed. Briefly, 2 µg genomic DNA was digested with 10 units of *Xmn*I for 1 h, 50 ng of linear DNA was religated with 5 units of T4 DNA ligase for 1 h at room temperature, and the sample was used as a template for amplification with *SMC6B*-specific primers. The resulting PCR product was cleaned with ExoSAP-IT™ PCR Product Cleanup Reagent (Thermo Fisher) and sequenced by Sanger sequencing.

### Root length assays and phenotypical analyses of mutant plants

Stratified, surface-sterilized seeds were evenly sown on square culture plates with half-strength MS medium with 0.8% (w/v) agar, and placed horizontally for 7 days. Subsequently, the seedlings were carefully pulled off the agar surface with tweezers and stretched onto fresh agar plates. Seedlings were photographed with a D90 digital camera (Nikon), and the length of the primary root was measured using the ImageJ plugin SmartRoot (Lobet et al., 2011). Detailed photographs were collected using an SZX16 binocular microscope equipped with a Regita 1,300 QImaging camera and QCapture × 64 software (both Olympus).

For sensitivity assays of single mutants, seeds were germinated on half-strength MS medium with 0.8% (w/v) agar containing individual chemicals: 20 μM zebularine (Z4775, Sigma-Aldrich), 20 nM (*S*)-(+)-camptothecin (CPT, C9911, Sigma-Aldrich), 10 μM ICRF-187 (D1446, Sigma-Aldrich), 100 nM, 10 μM and 100 μM AZD2461 (SML1858, Sigma-Aldrich), 100 nM, 100 μM and 1 mM 3-methoxybenzamide (M10050, Sigma-Aldrich), 100 μM, 1 and 4 mM 3-aminobenzamide (A0788, Sigma-Aldrich). Sensitivity to each chemical treatment in individual replicates was determined by calculating mean (treatment)/mean (mock). The experiment was performed as three biological replicates, each with at least 20 seedlings/replicate. The means of the three replicates are shown. Statistical significance was tested by one-way analysis ANOVA with post hoc Tukey HSD in R (Core R Team, 2020).

### Drug sensitivity assays

Drug sensitivity assays were performed as described (Dorn and Puchta, 2020). Stratified, surface-sterilized seeds were sown on culture plates with half-strength MS medium with 0.6% (w/v) agar, and cultivated for seven days. Subsequently, ten seedlings of each genotype were transferred to a six-well culture plate containing 5 ml of liquid half-strength MS medium (untreated control) or 4 ml of liquid half-strength MS medium (treated samples) per well under sterile conditions. The next day, 1 ml of genotoxin solution diluted in liquid half-strength MS medium was added to obtain the desired final concentration. Seedling fresh weight was measured after 13 days of exposure. Relative fresh weight was determined by comparison of fresh weight between treated and untreated samples for each genotype and concentration. The experiment was performed as three biological replicates, and the means of the three replicates are shown.

### Cell death analyses in roots

Seeds were sown on plates containing half-strength MS medium with 0.6% (w/v) agar and grown vertically for 5 days before transfer to liquid half-strength MS medium without (mock) or with 20 µM zebularine, 20 nM CPT or 10 µM ICRF-187 for 24 h. Afterwards, the seedlings were placed in 10 mg mL^–1^ propidium iodide (PI, Sigma) on slides and immediately analyzed and photographed using a Leica confocal microscope TCS SP8 (Leica, Wetzlar, Germany) and HC PL APO CS2 20x/0.75 DRY objective equipped by Leica LAS-X software with Leica Lightning module laser scanning confocal microscope (Leica). The pattern was checked in at least ten individual seedlings per treatment.

### Immunostaining and confocal microscopy

Immunostaining was performed as previously described (Prochazkova et al., 2022). Briefly, 5-day-old seedlings were incubated in 0 (mock) or 40 µM zebularine for 24 h. Seedlings were fixed with 4% (w/v) formaldehyde in Tris buffer (10 mM Tris-HCl pH 7.5, 10 mM NaEDTA, 100 mM NaCl) at 4°C for 20 min and washed 2 × 10 min with Tris buffer at 4°C. Seedlings were chopped in 500 µl LB01 buffer and filtered through 50-µm and 20-µm cell strainer caps. Flow cytometry analysis and sorting were carried out on a FACSAria II SORP flow cytometer and sorter (Becton Dickinson Immunocytometry Systems, San José, USA). The samples were analyzed at rates of 400–1,400 particles per second. Bivariate flow karyotypes of PI pulse area (PI-A) vs. DAPI pulse area (DAPI-A) fluorescence were acquired, and 20,000 events were recorded to create a bivariate flow karyotype for each experiment. Sorted regions were set on the flow karyotypes, and RFP-positive nuclei were sorted onto microscope slides with a 3-μl drop of PRINS buffer supplemented with 2.5% (w/v) sucrose (Kubaláková et al., 1997). Around 3,000 nuclei were sorted per slide. Slides were post-fixed with 4% (w/v) formaldehyde in phosphate-buffered saline (PBS) for 15 min and washed with PBS. For immunolocalization of SUMO1 and SUMO2/3, slides were incubated with a rabbit anti-SUMO1 or anti-SUMO3 primary antibody diluted 1:200 (ab5316 and ab5317, Abcam) at 4°C overnight, and a goat anti-rabbit Alexa Fluor 488-conjugated secondary antibody diluted 1:250 (A11008, Invitrogen) at room temperature for 2 h. The slides were shortly washed with 1× PBS, and nuclei were counterstained with DAPI (300 ng.µl^−1^) and mounted in Vectashield (H-1000, Vector Laboratories). Imaging was performed with a Leica confocal microscope TCS SP8 (Leica 265 Microsystems) and HC PL PAO CS2 63×/1.4 OIL objective equipped with Leica LAS-X software (Leica). Images were captured separately for each fluorochrome with 546-nm (MET1-RFP), 488-nm (Alexa Fluor 488), and 405-nm (DAPI) laser lines for excitation and appropriate emission filters. Processing of the final images and quantitative analysis of MET1-RFP colocalization with SUMO1 was performed in ImageJ using a fluorescent intensity profile for both correlated signals. The respective colocalization coefficients were calculated by Pearson's correlation coefficient in Microsoft Excel.

### Multiple sequence alignment

Multiple sequence alignment was performed to elucidate the conservation level of mutations in SMC6B. Sequence data of the proteins used in Supplemental Figure 8 were retrieved from UniProt: Q9FII7 (Arabidopsis SMC6B), Q9FLR5 (Arabidopsis SMC6A), D7MV22 (*Arabidopsis lyrata* SMC6B), P53692 (*Schizosaccharomyces pombe* SMC6), Q12749 (*Saccharomyces cerevisiae* SMC6), Q96SB8 (*Homo sapiens* SMC6), Q8GU52 (*Oryza sativa* SMC6), A0A0Q3L328 (*Brachypodium distachyon* SMC6), M4D8Z6 (*Brassica rapa* SMC), R0G894 (*Capsella rubella* SMC), A0A1S3ZHR2 (*Nicotiana tabacum* SMC6), A0A3Q7GL50 (*Solanum lycopersicum* SMC6), A0A2K2B516 (*Populus trichocarpa* SMC6), D7U753 (*Vitis vinifera* SMC6). Analyses were performed in the AliView program (Larsson, 2014).

### Protein structure analysis

The model of AtSMC6B subunit (UniProt ACC: Q9FIIH) was built using SWISS-MODEL (Bienert et al., 2017; Waterhouse et al., 2018). ScSMC6 (PBDID: 7qcd) (Hallett et al., 2022) was used as a template. More detailed hinge models were generated using AlphaFold2. All models were further processed in PyMOL (Schrödinger, 2015).

### Statistical methods

To determine statistically significant effects, one-way analysis of variance (ANOVA) with post hoc Tukey HSD (*P* ≤ 0.05) tests were performed in R (Core R Team, 2020). Statistical significance of colocalization between MET1-RFP and SUMO1 in the wild-type, *smc6b-1,* and *nse2-2* backgrounds was calculated with a chi-square test.

## Supplementary Material

koad020_Supplementary_Data

## Supplemental data

The following materials are available in the online version of this article.

**Supplemental Figure S1**. Design of the *HYPERSENSITIVE TO ZEBULARINE* (*HZE*) screen.

**Supplemental Figure S2.** Mapping-by-sequencing of *hze1-1*.

**Supplemental Figure S3**. Complementation crosses of selected *hze1* candidates.

**Supplemental Figure S4**. Validation of the translocation in *hze1-3*.

**Supplemental Figure S5**. Sensitivity of mutants in DNA–protein cross-link repair factors to DNA–protein crosslinking drugs.

**Supplemental Figure S6**. Sensitivity of WT and *smc6b-1* to potential DNA–protein crosslinking drugs. Representative growth phenotypes of WT and *smc6b-1* germinated on increasing concentrations of AZD2461 (AZD), 3-methylbenzamide (3-MB) or 3-aminobenzamide (3-AB).

**Supplemental Figure S7**. Immunolabelling of zebularine-treated nuclei from WT root nuclei stained with anti-SUMO1 (top panel) and anti-SUMO3 (bottom panel) antibody.

**Supplemental Figure S8**. Multiple protein sequence alignment analysis of SMC6B homologs from different species at the position of mutated amino acid residues in *hze1-1*.

**Supplemental Table S1**. Source data for the statistical analyses to support Figure 1.

**Supplemental Table S2**. Source data and statistical analyses for Figure 2, A and B, C.

**Supplemental Table S3**. Source data and statistical analyses for Supplemental Figure 5A, 5B, 5C.

**Supplemental Table S4**. Source data and statistical analyses for Figure 3A.

**Supplemental Table S5**. Source data for statistical analyses used in Figure 4A.

**Supplemental Table S6**. Source data of MET1-RFP colocalization with SUMO1 in WT, *smc6b-1* and *nse2-2* root nuclei after zebularine treatment.

**Supplemental Table S7**. Primers used in this study.

**Supplemental File S1**. Multiple protein sequence alignment shown in Supplemental Figure 8.
