# Correction to: SMC5/6 complex-mediated SUMOylation stimulates DNA–protein cross-link repair in Arabidopsis

**DOI:** 10.1093/plcell/koad120

**Published:** 2023-05-09

This is a correction to: Eva Dvořák Tomaštíková, Klara Prochazkova, Fen Yang, Jitka Jemelkova, Andreas Finke, Annika Dorn, Mahmoud Said, Holger Puchta, Ales Pecinka, SMC5/6 complex-mediated SUMOylation stimulates DNA–protein cross-link repair in Arabidopsis, *The Plant Cell*, Volume 35, Issue 5, May 2023, Pages 1532–1547, https://doi.org/10.1093/plcell/koad020

In the originally published version of this manuscript, the final author, Ales Pecinka, was incorrectly associated with the Functional Genomics and Proteomics, National Centre for Biomolecular Research (NCBR), Faculty of Science, Masaryk University, Kamenice 5, 62500 Brno, Czech Republic.

This error has been corrected online.

