# Supplementary material for: SMC5/6 complex-mediated SUMOylation stimulates DNA–protein cross-link repair in Arabidopsis
Source: Plant Cell. 2023 Jan 27;35(5):1532–47. doi: 10.1093/plcell/koad020 (PMC10118267; doi:10.1093/plcell/koad020)
Supplement: koad020_Supplementary_Data [file koad020_supplementary_data.zip › TPC2022RA01135D_Supplemental Figures and Tables.pdf]

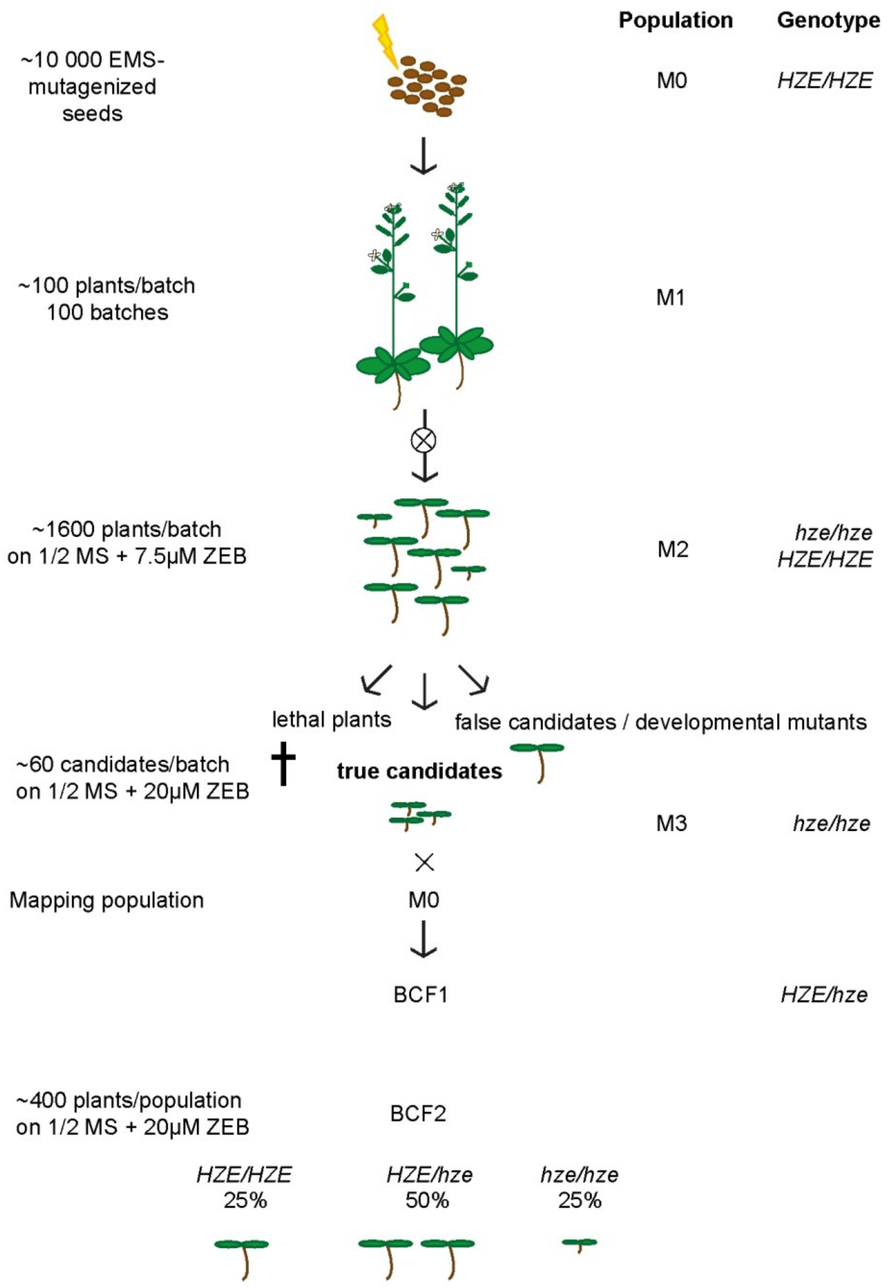

Supplemental Figure S1 Design of the *HYPERSENSITIVE TO ZEBULARINE* (*HZE*) screen. (Supports Figure 1)

Approximately 10,000 M0 WT Col-0 (zebularine-resistant) seeds were EMS-mutagenized. M1 seeds were sown onto soil as 100 batches, grown and M2 seeds were harvested for screening. Each batch was screened for zebularine-sensitive (ZEB^S, small) seedlings. The identified ZEB^S seedlings were propagated to the M3 generation and validated on mock and 20 μM zebularine-containing media. To identify the causal mutations for the ZEB^S phenotype, mapping populations were produced from a backcross to the parental line (BCF1) by self-pollination (BCF2). Approximately 100 ZEB^S seedlings were pooled in the BCF2 generation for mapping-by-sequencing.

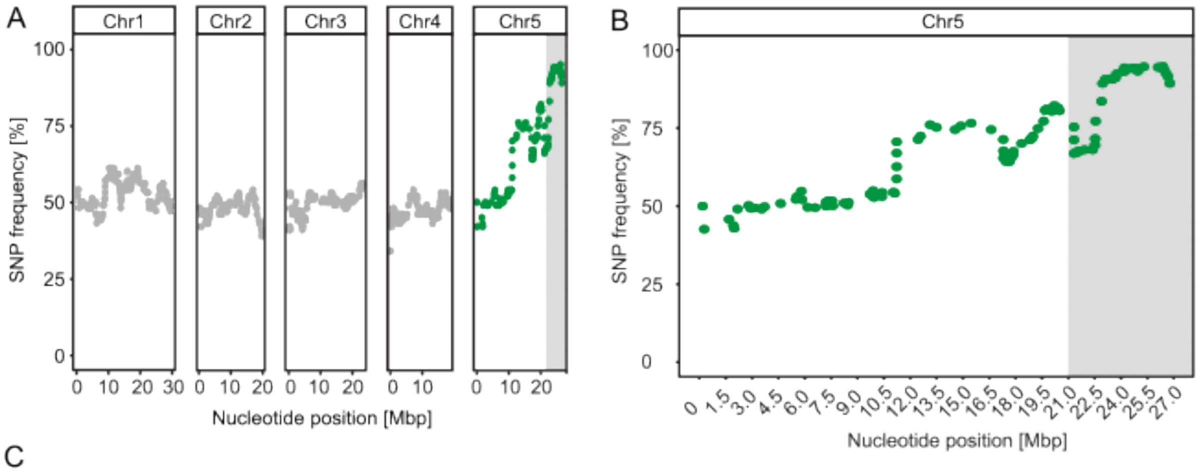

C

CHROMOSOME	NUCLEOTIDE	REF	ALT	PHREQ	PHRED	EFFECT	IMPACT	AA CHANGE	GENE ID	GENE NAME
Chr5	20497883	C	T	77	221	downstream_gene_variant	MODIFIER		AT5G50345	ESFL11
Chr5	21486698	C	T	85	219	synonymous_variant	LOW	S171S	AT5G53000	TAP46
Chr5	21538129	A	G	22	8	missense_variant	MODERATE	S618P	AT5G53130	CNGC1
Chr5	21571805	C	T	88	219	5_prime_UTR_variant	MODIFIER		AT5G53180	ATPTB2
Chr5	21583503	C	T	92	201	intron_variant	MODIFIER		AT5G53200	TRY
Chr5	21636865	C	T	83	220	missense_variant	MODERATE	R465Q	AT5G53320	AT5G53320
Chr5	21929792	C	T	73	198	missense_variant	MODERATE	L69F	AT5G54043	AT5G54043
Chr5	22248337	C	A	50	23	downstream_gene_variant	MODIFIER		AT5G54770	THI1
Chr5	22390787	C	T	92	228	downstream_gene_variant	MODIFIER		AT5G55180	AT5G55180
Chr5	22524150	C	T	87	219	stop_gained	HIGH	W141*	AT5G55600	AT5G55600
Chr5	22558309	C	T	91	226	splice_region_variant&intron_variant	LOW		AT5G55730	FLA1
Chr5	22679500	T	C	18	3	intron_variant	MODIFIER		AT5G56000	HSP90-4
Chr5	22876952	G	T	17	8	splice_acceptor_variant&intron_variant	HIGH		AT5G56500	CPN60B3
Chr5	22898537	C	T	100	188	upstream_gene_variant	MODIFIER		AT5G56555	AT5G56555
Chr5	22958383	C	T	93	228	upstream_gene_variant	MODIFIER		AT5G56750	NDL1
Chr5	23114014	C	T	100	228	splice_acceptor_variant&intron_variant	HIGH		AT5G57110	ACA8
Chr5	23473951	C	T	91	228	missense_variant	MODERATE	R16Q	AT5G57990	UBP23
Chr5	23506744	C	T	89	219	intron_variant	MODIFIER		AT5G58090	AT5G58090
Chr5	23687164	C	T	88	219	missense_variant	MODERATE	A1014T	AT5G58610	AT5G58610
Chr5	23771838	C	T	88	219	intron_variant	MODIFIER		AT5G58870	FTSH9
Chr5	24113756	C	T	100	228	missense_variant	MODERATE	R62C	AT5G59860	AT5G59860
Chr5	24201307	C	T	100	228	5_prime_UTR_variant	MODIFIER		AT5G60100	PRR3
Chr5	24232637	C	T	89	219	downstream_gene_variant	MODIFIER		AT5G60180	APUM19
Chr5	24594289	C	T	100	228	intron_variant	MODIFIER		AT5G61140	BRR2C
Chr5	24718215	C	T	100	228	missense_variant	MODERATE	D513N	AT5G61460	SMC6B
Chr5	24761443	C	T	94	228	intron_variant	MODIFIER		AT5G61580	PFK4
Chr5	24955160	C	T	90	219	missense_variant	MODERATE	E73K	AT5G62140	AT5G62140
Chr5	24981439	C	T	100	228	synonymous_variant	LOW	R459R	AT5G62190	RH7
Chr5	25128847	C	A	15	4	intron_variant	MODIFIER		AT5G62600	MOS14
Chr5	25999280	T	A	17	8	intron_variant	MODIFIER		AT5G65080	MAF5
Chr5	26119661	G	A	87	219	synonymous_variant	LOW	Y55Y	AT5G65350	HTR11
Chr5	26389422	G	A	90	220	missense_variant&splice_region_variant	MODERATE	P09S	AT5G65970	MLO10
Chr5	26408633	C	A	100	85	intron_variant	MODIFIER		AT5G66030	GRIP
Chr5	26454656	G	A	88	219	synonymous_variant	LOW	S316S	AT5G66200	ARO2
Chr5	26570035	G	A	88	219	missense_variant	MODERATE	E304K	AT5G66570	PSBO1
Chr5	26677708	G	A	77	221	missense_variant	MODERATE	G80R	AT5G66815	CEP5
Chr5	26785404	G	A	76	221	synonymous_variant	LOW	L187L	AT5G67110	ALC

Supplemental Figure S2 Mapping-by-sequencing of *hze1-1*. (Supports Figure 1)
A, Single nucleotide polymorphism (SNP) frequency plot based on the sequencing of genomic DNA from ~100 zebularine-sensitive BCF2 seedlings. Only SNPs with a PHRED score ≥ 100 were used. Individual points represent the average allele frequency of eleven consecutive SNPs (sliding window 11). The main candidate region on the bottom arm of chromosome 5 is highlighted by a gray background. B, Close-up

view of the SNP frequency plot at the position containing the candidate gene on chromosome 5 (in gray). C, List of candidate SNPs in the candidate region. The green background highlights the causal SNP at *SMC6B* (At5g61460). The associated SNP caused a missense mutation and was present in 100% (PHREQ) of all sequencing reads from the population of sensitive seedlings selected for the mapping. REF, nucleotide in reference sequence; ALT, alternative nucleotide; PHRED, probability score.

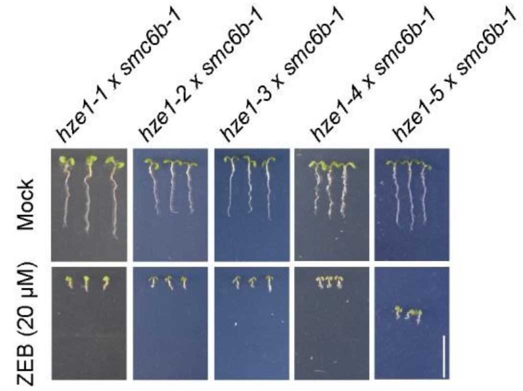

Supplemental Figure S3 Complementation crosses of selected *hze1* candidates. (Supports Figure 1) Representative growth phenotypes of F1 hybrid seedlings generated from complementation crosses with *smc6b-1*. See Figure 1A for the phenotype of the controls. Scale bar, 1 cm.

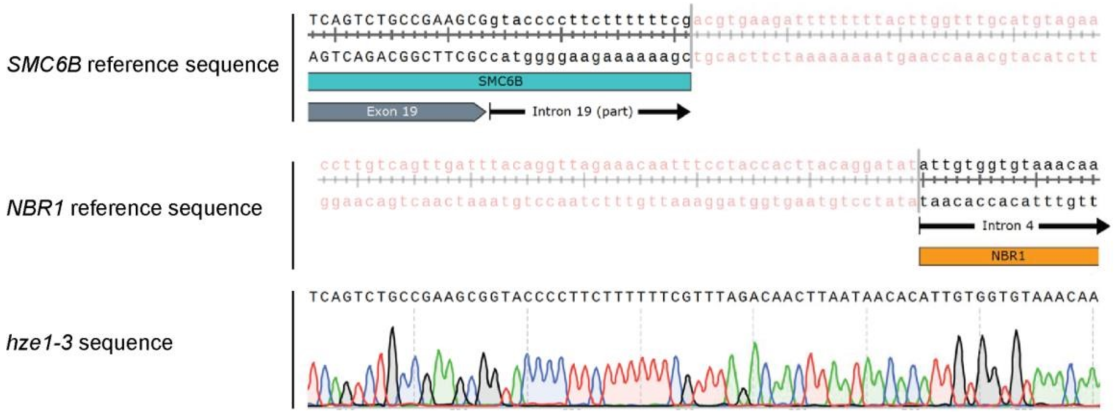

Supplemental Figure S4 Validation of the translocation in *hze1-3*. (Supports Figure 1) The translocation junction region was PCR amplified from *hze1-3* genomic DNA using a forward primer in *SMC6B* (chromosome 5) and a reverse primer in *NBR1* (chromosome 4), subjected to Sanger sequencing (bottom lane) and compared to the *SMC6B* (top lane) and *NBR1* (middle lane) reference sequences. Parts of reference sequences present are indicated by the black letters and the parts missing by partly shaded red letters. The junction region contains a unique filler sequence.

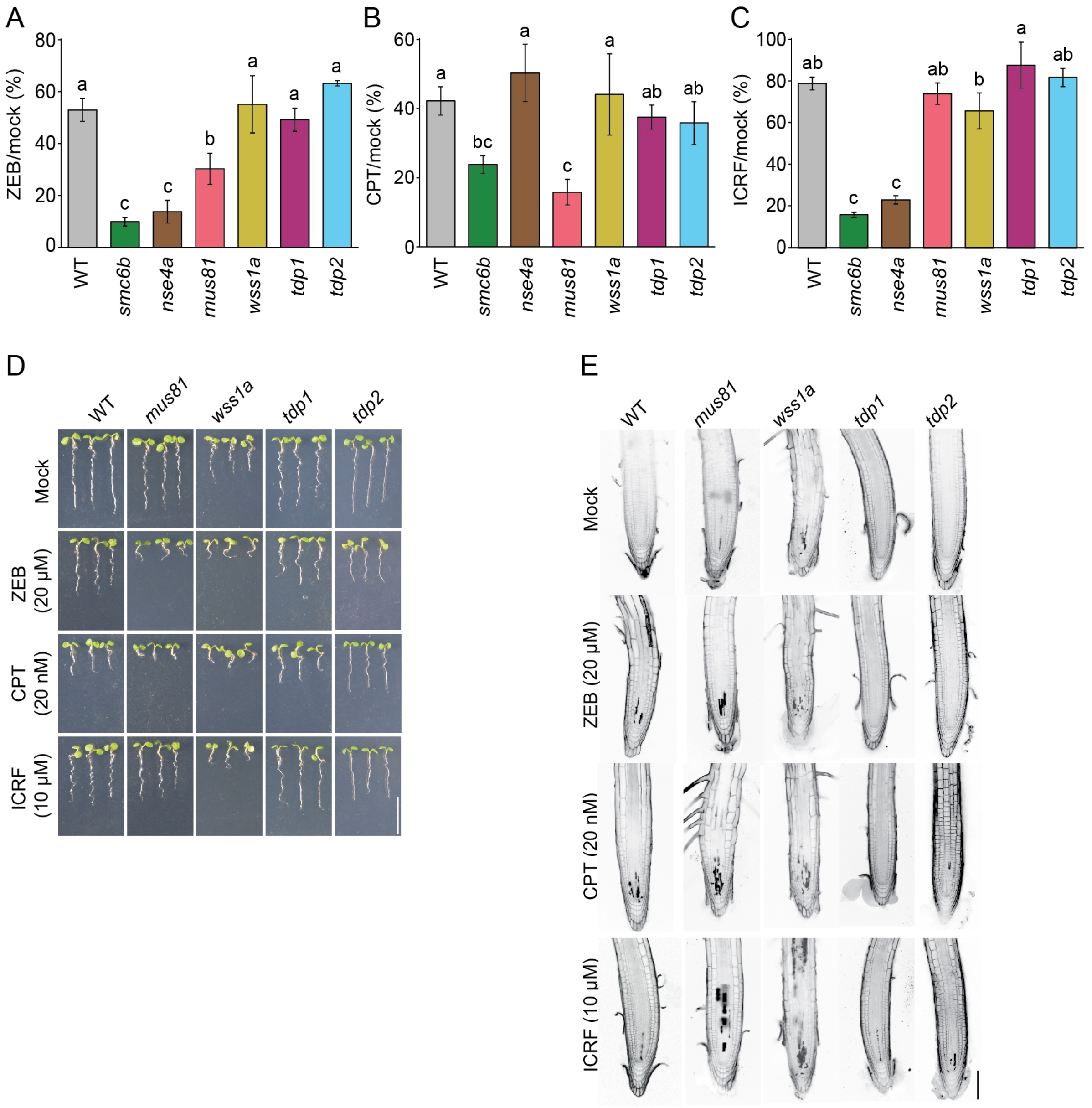

Supplemental Figure S5 Sensitivity of mutants in DNA-protein crosslink repair factors to DNA-protein crosslinking drugs. (Supports Figure 2)

A–C, Relative root length of wild type (WT) and mutants in DPC repair factors treated with 20 µM zebularine (ZEB), 20 nM camptothecin (CPT) or 10 µM ICRF-187 (ICRF) relative to mock conditions. Data are means ± SD from three biological replicates. Different lowercase letters indicate significant differences (P < 0.05) according to one-way ANOVA followed by Tukey's HSD test. The original experiment was split between Figures 1A, B, C, 2A, 2B, 2C and Supplemental Figures 5A, B, C, D. Therefore, these figures show identical images and data for the controls. D, Representative growth phenotypes of seedlings from (A). Scale bar, 1 cm. E, Representative confocal microcopy images of root tips stained with propidium iodide (PI), which labels dead cells (dark sectors). Five-day-old seedlings were treated for 24 h with 20 µM ZEB, 20 nM CPT, or 10 µM ICRF and then analyzed. Scale bar, 100 µm.

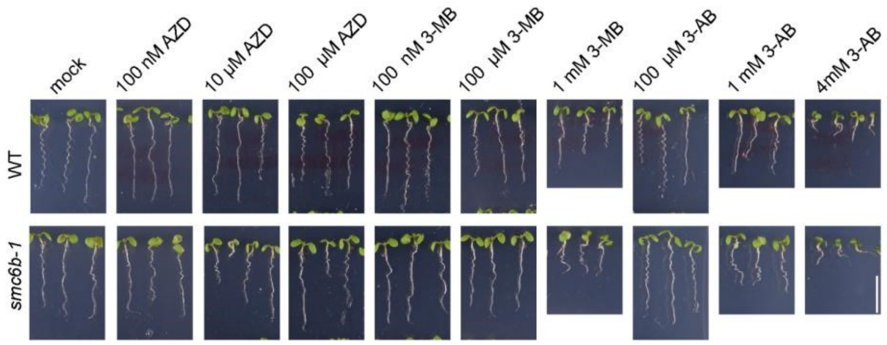

Supplemental Figure S6 Sensitivity of WT and *smc6b-1* to potential DNA-protein crosslinking drugs. (Supports Figure 2)

Representative growth phenotypes of WT and *smc6b-1* germinated on increasing concentrations of AZD2461 (AZD), 3-methylbenzamide (3-MB) or 3-aminobenzamide (3-AB). Scale bar, 1 cm.

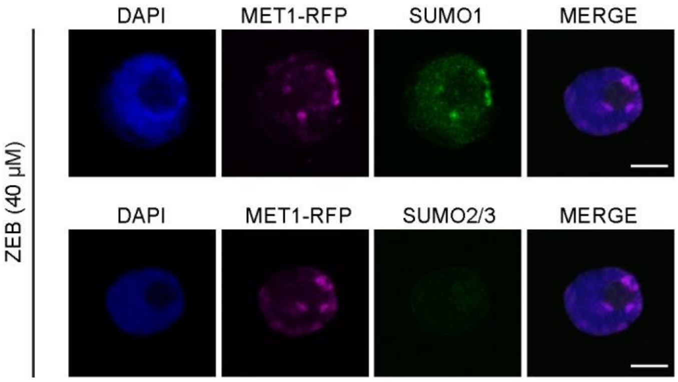

Supplemental Figure S7 Immunolabelling of zebularine-treated nuclei. (Supports Figure 5)

Nuclei are from WT root nuclei stained with anti-SUMO1 (top panel) and anti-SUMO3 (bottom panel) antibody. MET1-RFP signals were observed directly and nuclei were counterstained with DAPI. Scale bar, 5 µm.

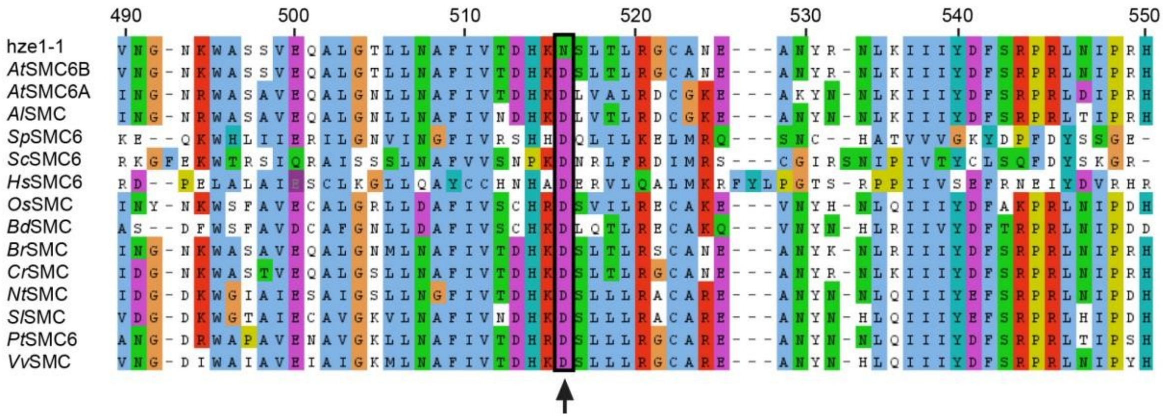

Supplemental Figure S8 Multiple protein sequence alignment analysis of SMC6B homologs from different species at the position of mutated amino acid residues in *hze1-1*. (Supports the Discussion) Multiple sequence alignment was performed using MUSCLE in the program AliView (Larsson, 2014). Numbers above the alignment indicate amino acid positions in *At*SMC6B. *hze1-1* – SMC6B mutant isolated from our genetic screen, *At – Arabidopsis thaliana*, *Al – Arabidopsis lyrata*, *Sp – Schizosaccharomyces pombe*, *Sc – Saccharomyces cerevisiae*, *Hs – Homo sapiens*, *Os – Oryza sativa*, *Bd – Brachypodium distachyon, Br – Brassica rapa*, *Cr – Capsela rubella*, *Nt – Nicotiana tabacum*, *Sl – Solanum lycopersicum*, *Pt – Populus trichocarpa*, *Vv – Vitis vinifera.* UniProt identifiers of proteins used for alignment are listed in the section Accession numbers of Material and methods. The amino acid position mutated in *hze1-1* is highlighted by the black frame and arrow.

Supplemental Table S1 Source data for the statistical analyses to support Figure 1.
Statistical significance tested with One-way ANOVA and is represented by adjusted P-value compared to respective WT control. As there is no statistical difference in both WT controls, there is only one WT presented in the graph.

ZEB/mock (%)

		**WT**	***smc6b-1***	***hze1-2***	***hze1-3***	**WT**	***hze1-1***	***nse4a-2***
Replicate 1		70.76	7.93	6.57	5.72	55.14	11.12	12.28
Replicate 2		60.22	8.27	9.74	8.47	68.52	10.83	17.59
Replicate 3		66.80	8.41	11.87	7.38	54.06	12.06	10.30
Replicate mean		65.93	8.20	9.39	7.19	59.24	11.34	13.39
SD		5.33	0.25	2.66	1.38	8.05	0.64	3.77
Statistical significance	WT		0.000	0.000	0.000	0.000	0.000	0.000
	smc6b-1			1.000	1.000		0.959	0.995
	hze1-1			0.996	0.866			0.995
	hze1-2				0.993			0.884
	hze1-3							0.535

Supplemental Table S2 Source data and statistical analyses for Figure 2A, 2B, 2C.
Experiment was performed in three biological replicates. Statistical significance was tested with One-way ANOVA (Confidence interval 95) with Tukey´s multiple comparison tests.

CPT/mock (%)

		**WT**	***smc6b-1***	***nse4a-2***	**WT**	***smc6b-1***	***hze1-1***	***hze1-2***	***hze1-3***
Replicate 1		39.44	20.74	41.70	40.12	22.90	25.70	17.91	18.09
Replicate 2		40.26	24.89	51.02	39.76	24.23	24.99	19.54	17.52
Replicate 3		46.89	25.64	58.27	43.27	21.09	22.76	19.62	19.02
Replicate mean		42.20	23.76	50.33	41.05	22.74	24.48	19.02	18.21
SD		4.08	2.64	8.30	1.93	1.57	1.54	0.97	0.76
Statistical significance	WT		0.00	0.17	1.00	0.00	0.00	0.00	0.00
	smc6b-1			0.00	0.00	1.00	1.00	0.90	0.77
	nse4a-2				0.17	0.00	0.00	0.00	0.00
	WT					0.00	0.00	0.00	0.00
	smc6b-1						1.00	0.73	0.57
	hze1-1							0.59	0.43
	hze1-2								1.00

ICRF/mock (%)

		**WT**	***smc6b-1***	***nse4a-2***	**WT**	***smc6b-1***	***hze1-1***	***hze1-2***	***hze1-3***
Replicate 1		82.36	16.07	20.78	60.02	19.62	25.64	15.92	13.07
Replicate 2		76.78	14.28	23.17	78.99	19.85	21.94	15.23	14.43
Replicate 3		77.29	16.68	24.75	93.00	19.68	26.74	15.75	17.26
Replicate mean		78.81	15.68	22.90	77.33	19.72	24.78	15.63	14.92
SD		3.08	1.25	2.00	16.55	0.12	2.51	0.36	2.14
Statistical significance	WT		0.00	0.00	1.00	0.00	0.00	0.00	0.00
	smc6b-1			1.00	0.00	0.99	0.97	0.99	0.97
	nse4a-2				0.00	0.82	1.00	0.82	0.75
	WT					0.00	0.00	0.00	0.00
	smc6b-1						0.62	1.00	1.00
	hze1-1							0.61	0.53
	hze1-2								1.00

Supplemental Table S3 Source data and statistical analyses for Supplemental Figure 5A, 5B, 5C. Average root length for each replicate in mm. The experiment was performed in three biological replicates. Statistical significance was tested with One-way ANOVA (Confidence interval 95%) with Tukey´s multiple comparison tests.

ZEB/mock (%)

		**WT**	***smc6b-1***	***nse4a-2***	***mus81-1***	**WT**	***tdp1-3***
Replicate 1		50.423	9.203	12.277	24.007	56.102	54.230
Replicate 2		58.039	11.750	18.700	35.920	52.100	47.215
Replicate 3		50.387	8.697	10.303	30.903	58.655	46.108
Replicate mean		52.950	9.883	13.760	30.276	55.619	49.184
SD		4.408	1.636	4.391	5.981	3.304	4.405
Statistical significance	WT		0.000	0.000	0.001	1.000	0.995
	smc6b-1			0.994	0.004		0.000
	nse4a-2				0.026		0.000
	mus81-1						0.008
	tdp1-3						
	wss1a-1						

		**WT**	***wss1a-1***	**WT**	***tdp2-5***
Replicate 1		48.895	44.136	56.635	64.412
Replicate 2		53.656	66.232	52.168	62.531
Replicate 3		61.251	55.016	53.269	62.737
Replicate mean		54.601	55.128	54.024	63.227
SD		6.232	11.048	2.327	1.032
Statistical significance	WT	1.000	1.000	1.000	0.517
	smc6b-1		0.000		0.000
	nse4a-2		0.000		0.000
	mus81-1		0.000		0.000
	tdp1-3		0.917		0.084
	wss1a-1				0.674

ICRF/mock (%)

		**WT**	***smc6b-1***	***nse4a-2***	***mus81-1***	***WT***	***tdp1-3***
Replicate 1		82.359	16.067	20.780	79.428	86.158	80.272
Replicate 2		76.785	14.280	23.173	69.463	87.604	82.169
Replicate 3		77.295	16.681	24.746	72.885	73.674	100.233
Replicate mean		78.813	15.676	22.900	73.925	82.478	87.558
SD		3.081	1.247	1.997	5.063	7.659	11.018
Statistical significance	WT		0.000	0.000	0.821	1.000	0.685
	smc6b-1			0.857	0.000		0.000
	nse4a-2				0.000		0.000
	mus81-1						0.163
	tdp1-3						
	wss1a-1						

		WT	***wss1a-1***	***WT***	***tdp2-5***
Replicate 1		83.618	55.727	82.107	83.758
Replicate 2		81.364	71.956	84.464	84.538
Replicate 3		79.544	69.071	78.956	76.600
Replicate mean		81.509	65.584	81.842	81.632
SD		2.041	8.658	2.764	4.375
Statistical significance	WT	1.000	0.043	1.000	1.000
	smc6b-1		0.000		0.000
	nse4a-2		0.000		0.000
	mus81-1		0.735		0.808
	tdp1-3		0.004		0.950
	wss1a-1				0.061

CPT/mock (%)

		**WT**	***smc6b-1***	***nse4a-2***	***mus81-1***	***WT***	***tdp1-3***
Replicate 1		39.440	20.737	41.700	11.681	43.214	39.273
Replicate 2		40.264	24.892	51.017	16.853	46.389	39.837
Replicate 3		46.890	25.639	58.266	18.873	37.923	33.498
Replicate mean		42.198	23.756	50.328	15.803	42.509	37.536
SD		4.084	2.641	8.304	3.709	4.277	3.509
Statistical significance	WT		0.022	0.762	0.001	1.000	0.983
	smc6b-1			0.001	0.782		0.156
	nse4a-2				0.000		0.224
	mus81-1						0.005
	tdp1-3						
	wss1a-1						

		WT	***wss1a-1***	***WT***	***tdp2-5***
Replicate 1		37.868	30.724	41.662	42.951
Replicate 2		45.036	52.729	41.099	31.610
Replicate 3		43.098	48.860	41.824	32.866
Replicate mean		42.001	44.104	41.528	35.809
SD		3.708	11.748	0.381	6.217
Statistical significance	WT	1.000	1.000	1.000	0.959
	smc6b-1		0.009		0.288
	nse4a-2		0.934		0.117
	mus81-1		0.000		0.011
	tdp1-3		0.912		1.000
	wss1a-1				0.742

Supplemental Table S4 Source data and statistical analyses for Figure 3A.
Average root length for each replicate in mm. Experiment was performed in three biological replicates. Statistical significance was tested with One-way ANOVA (Confidence interval 95%) with Tukey´s multiple comparison tests. Different letters in the graphs indicate significant differences (P < 0.05, one-way ANOVA followed by Tukey's test).

		WT	*smc6b-1*	*mus81-1*	*smc6b-1 mus81-1*
Replicate 1		1.466	1.315	1.527	0.378
Replicate 2		1.316	1.134	1.309	0.282
Replicate 3		1.331	1.101	1.401	0.283
Replicate mean		1.371	1.183	1.412	0.314
SD		0.083	0.115	0.109	0.055
Statistical significance	*smc6b-1*	0.143			
	mus81-1	0.946	0.067		
	smc6b-1 mus81-1	0.000	0.000	0.000	

		WT	*smc6b-1*	*wss1a-1*	*smc6b-1 wss1a-1*
Replicate 1		1.265	0.901	0.388	0.127
Replicate 2		1.464	1.007	0.479	0.126
Replicate 3		1.262	0.957	0.425	0.148
Replicate 4		1.494	1.201	0.396	0.162
Replicate 5		1.395	1.182	0.513	0.166
Replicate mean		1.376	1.049	0.440	0.146
SD		0.109	0.135	0.054	0.019
Statistical significance	*smc6b-1*	0.000			
	wss1a-1	0.000	0.000		
	smc6b-1 wss1a-1	0.000	0.000	0.001	

Supplemental Table S5 Source data for statistical analyses used in Figure 4A.
Relative fresh weight of *smc6b-1 mus81-1, smc6b-1 wss1a*-1 double mutants and the respective single mutants and WT plants, relative to untreated control after treatment with ZEB. Statistical significance was tested with One-way ANOVA (Confidence interval 95%) with Tukey´s multiple comparison tests. Different letters in the graphs indicate significant differences (P < 0.05, one-way ANOVA followed by Tukey's test). To increase statistical power of some highly variable samples, the experiment was performed in at least three biological replicates.

**Zebularine/mock (%)**			
		20 µM	5 µM
Replicate 1	WT	83.660	92.026
	smc6b-1	28.280	65.761
	wss1a-1	66.638	86.424
	wss1a-1 smc6b-1	22.622	74.681
Replicate 2	WT	83.872	94.251
	smc6b-1	40.177	64.952
	wss1a-1	83.492	97.354
	wss1a-1 smc6b-1	22.273	35.927
Replicate 3	WT	77.636	85.683
	smc6b-1	28.343	59.885
	wss1a-1	58.505	70.948
	wss1a-1 smc6b-1	21.643	46.726
Replicate mean	WT	81.723	90.653
	smc6b-1	32.267	63.533
	wss1a-1	69.545	84.909
	wss1a-1 smc6b-1	22.179	52.444
SD	WT	3.541	4.446
	smc6b-1	6.851	3.185
	wss1a-1	12.745	13.268
	wss1a-1 smc6b-1	0.496	20.000
Statistical significance compared to WT	WT		
	smc6b-1	0.000	0.020
	wss1a-1	0.264	0.826
	wss1a-1 smc6b-1	0.000	0.001
Statistical significance compared to *smc6-1*	WT	0.000	0.020
	smc6b-1		
	wss1a-1	0.001	0.058
	wss1a-1 smc6b-1	0.402	0.078
Statistical significance compared to *wss1a-1*	WT	0.264	0.826
	smc6b-1	0.001	0.058
	wss1a-1		
	wss1a-1 smc6b-1	0.000	0.003
Statistical significance compared to *smc6b-1 wss1a-1*	WT	0.000	0.001
	smc6b-1	0.000	0.078
	wss1a-1	0.000	0.003
	wss1a-1 smc6b-1		

ZEB/mock (%)			
		20 µM	5 µM
Replicate 1	WT	73.362	89.391
	smc6b-1	27.766	56.774
	mus81-1	50.625	55.328
	smc6b-1 mus81-1	24.030	35.169
Replicate 2	WT	71.241	89.468
	smc6b-1	35.161	66.257
	mus81-1	55.062	69.851
	smc6b-1 mus81-1	34.511	49.907
Replicate 3	WT	82.105	96.140
	smc6b-1	32.492	70.347
	mus81-1	56.289	74.528
	smc6b-1 mus81-1	42.623	55.738
Replicate 4	WT		82.257
	smc6b-1		53.067
	mus81-1		59.071
	smc6b-1 mus81-1		40.088
Replicate 5	WT		94.451
	smc6b-1		53.780
	mus81-1		59.331
	smc6b-1 mus81-1		40.602
Replicate mean	WT	75.570	90.342
	smc6b-1	31.807	60.045
	mus81-1	53.992	63.622
	smc6b-1 mus81-1	33.721	44.301
SD	WT	5.759	5.669
	smc6b-1	3.745	8.048
	mus81-1	2.980	8.994
	smc6b-1 mus81-1	9.322	9.309
Statistical significance compared to WT	WT		
	smc6b-1	0.000	0.000
	mus81-1	0.010	0.000
	smc6b-1 mus81-1	0.000	0.000
Statistical significance compared to *smc6-1*	WT	0.000	0.000
	smc6b-1		
	mus81-1	0.008	0.874
	smc6b-1 mus81-1	0.978	0.021
Statistical significance compared to *mus81-1*	WT	0.010	0.000
	smc6b-1	0.008	0.874
	mus81-1		
	smc6b-1 mus81-1	0.014	0.004
Statistical significance compared to *smc6b-1 mus81-1*	WT	0.000	0.000
	smc6b-1	0.978	0.021
	mus81-1	0.014	0.004
	smc6b-1 mus81-1		

Supplemental Table S6 Source data of MET1-RFP colocalization with SUMO1 in WT, *smc6b-1* and *nse2-2* root nuclei after zebularine treatment. (Supports Figure 5)

	MET1-RFP			*smc6b-1* MET1-RFP		
	SUMO			SUMO		
	colocalized	non-colocalized	total	colocalized	non-colocalized	total
Replicate 1	174	26	200	25	75	100
Replicate 2	45	19	64	12	55	67
Replicate 3	76	14	90	5	18	23
Total	295	59	354	42	148	190

	nse2-2 MET1-RFP		
	SUMO		
	colocalized	non-colocalized	total
Replicate 1	22	73	95
Replicate 2	24	86	110
Replicate 3	25	67	92
Total	71	226	297

Supplemental Table S7: Primers used in this study.

Target	Primer name	Sequence 5' to 3'
SMC6B	ET0035	AGCTTCAACGTGAAATCATGG
SMC6B	ET0036	CTAGACAACATGTCATACCGGG
T-DNA SALK	LB_AP1	ACTGGAACAACACTCAACCCTATCT
MUS81	ET0222	TTTCTTGTTTGAGGTGCCCTTTTT
MUS81	ET0223	ATTGATTCATACCCAACAGGAGCG
T-DNA GABI-kat	ET0088	ATAATAACGCTGCGGACATCTACATTTT
WSS1A	ET0240	TTGCATCGAAGATCGGGAAGG
WSS1A	ET0241	GCGTTATGAGGACCATGAGCG
TDP1	ET0246	CACTTGTCTTCCTTAATGGCTC
TDP1	ET0247	GCTTGAAGAAATGGTGACCAGGG
TDP1	ET0248	GACACTTGTCTTCCTCAGG
SMC6B	smc6b_product1_fw	CCCTAAGCAGTCGCATCAAT
SMC6B	smc6b_product1_rs	CCACACACGGATTTTCAACA
SMC6B	smc6b_product2_fw	AATGTGAGTTTTGGCCGTTC
SMC6B	smc6b_product2_rs	TTCATTGCAGTTGATTCATCC
SMC6B	smc6b_product3_fw	TCGAGCTCAGACTGGTTGATT
SMC6B	smc6b_product3_rs	TTACCACATGGGAGCCAATAG
SMC6B	smc6b_product4_fw	ACATGCTCACCAGGTTACTGC
SMC6B	smc6b_product4_rs	TGCTTCGATCTCAGCAGCGA
SMC6B	smc6b_product5_fw	CGCTAGTATCCATAACCGTT
SMC6B	smc6b_product5_rs	GAGCGTTGAATCTGCTTAGAA
SMC6B	smc6b_product6_fw	GGCAGTAAGAGTCGCTTTCC
SMC6B	smc6b_product6_rs	ATGGACATTTCTTAATCCGAAGC
SMC6B	ET0276	AAAGAAGATTGAGAAAGACC
NBR1	ET0372	AGGGCAATCAGCTGTTGCC
