# Supplementary material for: SMC5/6 complex-mediated SUMOylation stimulates DNA–protein cross-link repair in Arabidopsis
Source: Plant Cell. 2023 Jan 27;35(5):1532–47. doi: 10.1093/plcell/koad020 (PMC10118267; doi:10.1093/plcell/koad020)
Supplement: koad020_Supplementary_Data [file TPC2022RA01135D_PeerReviewReport.pdf › TPC2022RA01135D_PeerReviewReport.pdf]

SMC5/6 complex-mediated SUMOylation stimulates DNA-protein crosslink repair in Arabidopsis

Eva Dvořák Tomaštíková, Klara Prochazkova, Fen Yang, Jitka Jemelkova, Andreas Finke, Annika Dorn, Mahmoud Said, Holger Puchta, Ales Pecinka

Corresponding author: Ales Pecinka (pecinka@ueb.cas.cz), Eva Dvořák Tomaštíková (tomastikova@ueb.cas.cz).

Review timeline:

TPC2022-RA-00068	Submission received:<br>1st Decision:	Jan. 18, 2022<br>Mar. 22, 2022 *manuscript declined*
**TPC2022-RA-01135**	Submission received:<br>1st Decision:<br>Final acceptance:	Nov. 21, 2022<br>Dec. 20, 2022 *acceptance pending, sent to science editor*<br>Jan. 23, 2023

REPORT: (The report shows the major requests for revision and author responses. Minor comments for revision and miscellaneous correspondence are not included. The original format may not be reflected in this compilation, but the reviewer comments and author responses are not edited, except to correct minor typographical or spelling errors that could be a source of ambiguity.)

TPC2022-RA-00068 1st Editorial decision – *declined* Mar. 22, 2022

First, we sincerely apologize for the length of time that it took to get a decision back to you. We had one particularly tardy reviewer, and this delayed the process. We typically aim to get comments back in 30-40 days or so.

Thank you for choosing to send your manuscript entitled "SMC5/6 complex is a key player in DNA-protein crosslink repair in Arabidopsis" for consideration at The Plant Cell. Your submission has been evaluated by members of the editorial board as well as expert reviewers in your field, and we regret to inform you that we are not able to recommend publication of this manuscript in its current form. We have not made this decision lightly. We have had input from multiple scientists, and we have solicited post-review comments as well.

That said, during the post-review consultation session, we also agreed that if you could address the major points raised by the reviewers by new experiments, we would welcome a resubmission. This may be treated as a new submission, but we would attempt to use at least some of the same reviewers. Nevertheless, reviewers may be asked to assess as a new manuscript (i.e. are the claims fully supported by the data and do the results presented move the field forward?), and not only whether previous reviewer comments have been addressed.

Your manuscript was read by three referees. You will see that there was a consensus that the work is solid and interesting showing the role of the SMC5/6 complex in the repair of DPCs damages. Reviewer1 was more supportive (but also had several comments and suggestions) while reviewers 2 and 3 had more reservations and thought that the manuscript has only a limited novelty and at times is quite speculative. Under this situation, the work would be acceptable only if you can provide additional data that would strengthen the structural aspects (see comments of reviewer2) and also if you can provide experimental data regarding SUMOylation that would make the model less speculative than it currently is. Providing additional findings on other candidates of the screen might also strengthen the manuscript as well as addressing all the other points raised by the referees.

-- Reviewer comments:

[Reviewer comments shown below along with author responses]

TPC2022-RA-01135 Submission received Nov. 21, 2022

Reviewer comments on previously declined manuscript and **author responses:**

<u>Reviewer #1:</u>

The manuscript by Tomaštíková et al describes the role of conserved Structural maintenance of chromosomes SMC5/6 complex in DPC repair. The authors perform a forward-directed genetic screen to identify new factors involved in the repair of DPCs and have found several alleles of SMC5/6 complex core subunit, SMC6B. They show that the SMC5/6 complex is an important factor involved in the repair of several types of DPCs that is involved both in the nucleolytic and proteolytic DPC repair, together with MUS81 and WSS1A factors respectively.
The manuscript is well written, and the data are adequately presented and support the conclusions drawn by the authors. The experiments have been carefully performed, and interpretations of data are sound. This work presents results of high interest that should be useful to a wide audience of researchers in the field, and provides good evidence supporting the implication of a new player (SMC5/6 complex) in the repair of DPCs.
I have several comments for a revised version.

Response: We thank reviewer #1 for the positive evaluation of our work. Below, are the point-by-point responses to all the concerns.

The relative root length of tpd2 mutants is missing in figure S5. Is there a correlation between the relative root length of tpd2 mutants and the dead cells measured with root tips stained with propidium iodide? The authors must comment.

Response: There was a mismatch in the figure. The new version of Figure S5 now includes the root length and propidium iodide staining of the *tdp2* mutant. This presumably null *tdp2* allele (CRISPR/Cas9 line with a 5-bp deletion in 1st exon of TDP2 resulting in a frameshift) is insensitive to the chemical treatments. This correlates with an absence of dead cells in its root tips.

Are the mutants *hze1-1* (mutation located at SMC flexible hinge), *hze1-2* (mutation mapping at the ATPase) and *hze-2-3* (translocation) equally sensitive to CPT and ICRF than *smc6b-1*? Could the author explain why they have carried out the subsequent characterization only with *smc6b-1*.

Response: We now included sensitivity experiments and cell dead assay with the *hze1* alleles on CPT and ICRF (Figure 2). We apologize for not including this already in the first version of our manuscript. As apparent, the mutant phenotypes of all EMS alleles are not statistically significantly different from the T-DNA insertional mutant allele *smc6b-1*.

We performed most of the other experiments with *smc6b-1* because the EMS mutant lines contain many other mutations and these mutants might cause additive sensitivity or even segregate for lethal mutations. During the revision, we started preparing also backcrosses to WT that will remove most of the background mutations and can be used for future experiments. We agree that the *hze1-1* (D513N) mutant can be of potential interest to the community studying the structure and functions of the SMC5/6 complex.

Figure 4A: The difference in fresh weight of the single mutants as compared to the double mutants treated with 5 µM zebularine is statistically significative and support the conclusion of a greater sensitivity of the double mutant to ZEB. But the differences found are not so high to support the conclusion stated at line 250 of a hyper-sensitivity for the double mutant.

Response: We agree and changed the statement to „increased sensitivity“.

Figure 4 and Figure 6. The root tips stained with propidium iodide at 5 µM are not shown. This data could help to explain the, apparently contradictory results found in the treatment with ZEB, where the higher sensitivity of the double mutant can be only appreciated at the lower dose.

Response: We thank the reviewer for this comment. The fresh weight assay is very sensitive in the means of concentration range. Therefore, we always use a gradient of concentrations from low to high. The amount of cell death is less restrictive for the concentration range and the sensitivity effect is usually visible even with the standard (high) concentrations used (20 µM ZEB).

The sentence starting in line 163 "Further screening identified two additional ZEB hypersensitive but otherwise phenotypically WT-like candidates, which is characteristic l for *smc6b* mutant" do not have sense.

Response: The sentence has been modified as follows: „To reveal whether there are other *HZE1* mutant alleles in

our collection, we analyzed phenotypes of the remaining (in the meantime selected) candidates on zebularine and found three additional zebularine hypersensitive but otherwise phenotypically WT-like candidates. We performed complementation crosses of these candidates with *smc6b-1* followed by zebularine sensitivity assays, which suggested that they are all allelic (Supplemental Figure S3).". For information, we added a new mutant allele retrieved from the screen during the work on the manuscript.

Line 226. "The SMC6B and MUS81 act in parallel pathways in DPC repair". Delete "The"

Response: Deleted.

Line 264: The sentence "Double mutant of SMC6B with WSS1A confirm the role of SMC5/6 in DPC repair" should be rephrased.

Response: We introduced number of changes in the manuscript and this sentence was removed.

Line 368: "Combing loss of WSS1A with mutants 369 of NHEJ result in severe growth and DCP repair deficiencies." should be changed to "Combination of WSS1A loss with mutations in NHEJ results in severe growth and DCP repair deficiencies", or a similar sentence.

Response: The sentence was changed as suggested.

Figure 2 legend: The sentence "SMC5/6 complex repairs also type 2 and 4 DNA protein crosslinks" should be "SMC5/6 complex repairs also type 3 and 4 DNA protein crosslinks".

Response: The sentence was changed as suggested.

Reviewer #2:

The work describes the isolation of mutant alleles of the structural maintenance of chromosomes 6B (SMC6B) gene as being causative for a hypersensitivity phenotype towards zeburaline, a drug triggering DNA-protein crosslinks (DPCs). Subsequently, by comparing drug sensitivity of single versus double mutants, SMC6B's involvement in DPC repair is demonstrated to be specific for certain types of DPCs, as well as to act in a pathway being different from that of the MUS81 endonuclease and the WSSA protease.
Next to the identification of the mutant SMC6B alleles via a forward genetics screen, all data are extrapolated from genetic interaction, rather than biochemical assays. However, although limited in the range of applied techniques, the identification of SMC6B in the repair of DPCs is novel and covers a topic of DNA damage repair being barely studied in plants. The experiments are well described, and results are convincing.
Nevertheless, at least when being within reach, it would be nice to see this work being complemented at the structural (1) and the physiological level (2), as it would potentially broaden the interest in the obtained results.

Response: We thank reviewer #2 for this careful evaluation and for the constructive suggestions on how to improve our manuscript. Responses to specific comments are provided below.

1. These exists a nice structural model of SMC6B in the Alfafold Protein Structure Database (https://alphafold.ebi.ac.uk). It likely helps to describe and understand better the impact of the mutations identified in the hinge and ATPase domain.

Response: We thank reviewer #2 for this suggestion. We performed *At*SMC6B (UniProt ACC: Q9FIIH) homology modeling based on the SMC5/6 structure (PDBID: 7qcd) and compared the WT Arabidopsis SMC6B hinge region with the D513N mutated variant. The mutation is localized in one of the alpha helices of the Subdomain I of the SMC6B hinge domain. Careful checking of the literature did not reveal any specific function for this particular residue of the hinge domain. Based on the position inside of the hinge domain, D513 is most likely important for the proper folding of the hinge domain.

We included this information in the Results section, Figure 1F, 1G and Discussion.

The *hze1-4* carries mutation in the ATPase head domain that is responsible for the interaction with DNA.

2. I understand that a pharmacological approach is the easiest and most reproducible way to study the topic of interest (as being used in most DNA damage repair manuscripts), but could the authors test a specific abiotic stress condition that might trigger the same effects (and thus allowing to highlight the importance of the findings under more physiological conditions)?

Response: This is a very good comment and we would like to do such an experiment as soon as possible. However, we are not aware of natural environmental stress factors that would induce specifically DNA-protein crosslinks (i.e. without inducing other types of structurally different types of DNA damage). In case the reviewer #2 knows such stress(es), we will be grateful for his/her advice.

Minor comments:

One before the last sentence of abstract is unclear, as it does not explain MUS81 and WSS1A. Additional, 'type 1 DPCs' likely does not mean too much to most readers. I believe that there is room to make this abstract more accessible.

Response: The sentence was removed and abstract adapted based on the new data that we obtained during the recent round of experiments.

Only one (rather old) reference is given to highlight the effects of DPCs (Barker et al., 2005). Can other more recent papers be given?

Response: We added several more recent references to this point.

Isn't the suggested SUMOylation hypothesis not testable?

Response: We thank reviewer #2 for this comment. Based on it, we performed additional experiments focused on SUMO whose results supported our hypothesis. The results of these experiments are now provided in Figure 5 and Supplemental Figure 7 and in the Results section. We performed immunofluorescence labeling of SUMO1 and SUMO3 on flow-sorted MET1-RFP positive nuclei before and after zebularine treatment. After zebularine treatment, we observed strong colocalization of endogenous MET1-RFP with SUMO1 but not SUMO3, indicating that MET1-DPCs are SUMOylated. Next, we repeated the experiment in the *smc6b-1* and *nse2-2* (E3 SUMO ligase) mutant background and observed a significant decrease in the colocalization of SUMO1 and MET1-RFP after zebularine treatment. Thus, we provide direct evidence that the majority of the MET1-DPC SUMOylation is deposited in the SMC5/6 complex dependent manner. We also tested the SUMO levels after zebularine treatment with western blotting. However, we did not observe any changes, probably because only low amount of cells contain high amount of zebularine-induced DNA-protein crosslinks (about 2% based on our flow-cytometric data).

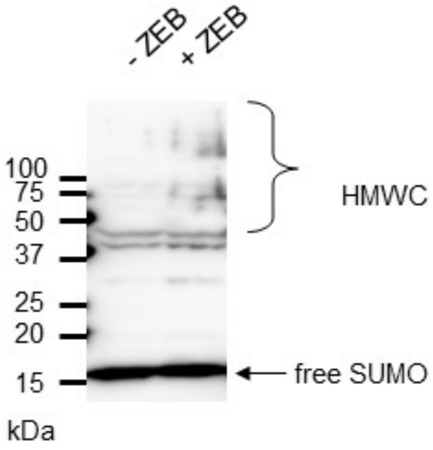

Western blot analysis of SUMO1 levels upon zebularine treatment. 7 days old wild-type seedlings were treated with either mock or 40 µM zebularine for 24 hrs. Approximately 200 mg of seedlings were ground in liquid nitrogen. Total proteins were extracted in Extraction buffer (0.15 M NaCl, 0,05 M Tris-HCl pH 7.5, 0,1% Tween20, 10% glycerol, 1 mM DTT, 1 mM Pefabloc SC Roche, 1x Protease Inhibitor Cocktail Roche) for 10 min on ice. Extracts were centrifuged at 14000 rpm at 4°C for 40 min, mixed 3:1 with 4x Laemmli sample buffer (Bio-Rad), boiled for 5min, and cleared by

centrifugation. The protein extracts were then separated by SDS-PAGE, transferred to polyvinylidene difluoride membranes. SUMOylated proteins were detected with αSUMO1-R primary antibody overnight (Abcam ab5316) and HRP-conjugated anti-rabbit secondary antibody for 2 hours. The immunoreaction was developed using the SuperSignal™ West Pico PLUS Chemiluminescent Substrate (Thermo Fisher 34580) and detected with Amersham Imager 600 (GE HealthCare Life Sciences LAS-3000 Luminescent Image.

TPC2022-RA-01135 1st Editorial decision – *acceptance pending* **Dec. 20, 2022**

We are pleased to inform you that your paper entitled "The SMC5/6 complex-mediated SUMOylation stimulates DNA-protein crosslink repair in Arabidopsis" has been accepted for publication in The Plant Cell, pending a final editorial review by a science editor. At this stage, your manuscript will be evaluated by a science editor with respect to its presentation of scientific content, compliance with journal policies, and presentation for a broad readership.

Final acceptance from Science Editor **Jan. 23, 2023**
